# Can you read my poker-face? Adapting the still-face paradigm to explore dog’s interspecific communication

**DOI:** 10.1007/s10071-026-02059-z

**Published:** 2026-03-14

**Authors:** Chiara Canori, Giulia Pedretti, Tiziano Travain, Chiara Annoni, Laura Sabbadini, Paola Valsecchi

**Affiliations:** 1 Department of Medicine and Surgery, University of Parma, Via Gramsci 14, Parma, 43126 Italy; 2 Department of Chemistry, University of Parma, Life Science and Environmental Sustainability, Viale delle Scienze 17/A, Parma, 43124 Italy; 3 Department of Biosciences, University of Milan, Via Celoria, 26, Milan, 20133 Italy; 4 Centro Cinofilo e Accademia di Formazione Ca’Nina, Desenzano del Garda, Località Montebruno, 25015 Italy

**Keywords:** Domestic dog, Interspecific communication, Still-face effect, Visual displays, Dog-human bond

## Abstract

Domestic dogs rely on a sophisticated repertoire of visual displays to regulate social interactions. However, the interpretation of some of these displays, such as facial expressions, remain unclear. In the present study, we investigated the communicative strategies dogs’ employ to re-establish a positive interaction with humans. We adopted a modified version of the classical Still-Face Paradigm and introduced two non-responsive phases: the classical “still-face” phase (human attentive but unresponsive) and a novel “face-away” phase (human turned away and inattentive) to test the flexibility of communicative signals modalities used by dogs. The test was conducted with both the owner (attachment figure) and a familiar human (familiar dog trainer). Results showed that dogs displayed a rich repertoire of visual and facial signals, which may serve affiliative and communicative functions, particularly during the first interaction and, overall, more towards their owner compared to the familiar human. Confirming a still-face effect, the frequency and duration of these behaviours significantly decreased in the non-responsive phases, compared to the first interaction. Contrary to our predictions, no differences emerged between the still-face and face-away phases and the exhibition of signals generally decreased in subsequent phases indicating a possible carry over effect. The higher communicative persistence towards the owner compared to the familiar human underscores the crucial role of the attachment bond in shaping the dog’s communicative strategies. These findings highlight the attachment bond as a key factor in interspecific communication, extend current research on the still-face paradigm in domestic dogs, and provide a valuable methodological tool for future investigations.

**Supplementary Information:**

The online version contains supplementary material available at 10.1007/s10071-026-02059-z.

## Introduction

Domestic dogs (*Canis familiaris*) rely on a sophisticated communication system to interact with conspecifics and regulate social interactions (Siniscalchi et al. 2018; Handelman 2012). Among the communicative modalities they use, visual signals have received increasing attention for their role in conveying emotional states and intentions in different social contexts (Bradshaw and Rooney 2016). For example, dogs display specific body and tail postures to communicate intentions and emotional states, such as modifying tail height and body stiffness during agonistic or affiliative interactions (Leaver and Reimchen 2008; van der Borg et al. 2015), exhibiting asymmetric tail wagging (right- vs. left-biased) in response to positive or negative social stimuli (Siniscalchi et al. 2013), or showing dynamic facial expressions (Kaminski et al. 2017; Caeiro et al. 2017). Furthermore, they engage in eye-gazing behaviours (Prato-Previde and Marshall-Pescini 2014) to request assistance when a reward is out of reach/inaccessible (Hare et al. 1998; Passalacqua et al.^ 2011^), or to indicate the location of a specific object or reward to a human partner (Miklósi et al. 2000).

Facial expressions appear to play a central role in canine communication and research suggests that these signals are influenced by the presence of a potential receiver (Pedretti et al. 2022) – an essential criterion for a behaviour to be considered communicative (Leavens and Hopkins 1998; Leavens et al. 2005). In fact, displays such as eye-blinking, lip-wiping, ears flattening occur at higher frequencies during a frustration context when a social partner (conspecific or human) is present compared to when no social partner is present (Pedretti et al. 2024).

Dogs’ interspecific interactions are often characterized by complex communicative exchange: dogs not only modify their visual signals based on the presence and attentiveness of a human audience (Pedretti et al. 2022, 2024; Gaunet and Deputte 2011) but also appear to adapt their signalling based on the context and intention of the receivers (Pedretti et al. 2023). However, the specific functions of these visual cues in human-directed communication remain unclear. In the last years, three studies used the still-face paradigm to investigate dog-human bond and communication (Barrera et al. 2021; Cavalli et al. 2023; Byrne et al. 2024). The still-face paradigm was developed by Tronick and colleagues (1975) and extensively used to study mother-infant communication as a two-way process (Adamson and Frick 2003 for an extensive review). The classical experiment by Tronick and colleagues (1978) consisted of (1) a positive interaction between the infant and their caregiver; (2) an abrupt break in the interaction in which the caregiver puts on a “still-face” and stopped responding to the child’s attempt to interact while maintaining eye contact (still-face phase); (3) a reunion phase, when the caregiver resumes the interaction and carries on the phase analogously to the first interaction. Infants typically showed increase physiological stress levels (increase in heart rate – Haley et al. 2006; cortisol levels – Haley and Stansbury 2003; Haley et al. 2006; and skin conductance – Ham and Tronick 2006) and stress-related behaviours (i.e., gaze aversion, high number negative of vocalizations, changing in facial expressions -such as less smiling- and body movements) caused by the abrupt break in social contact with a caregiver, violating the infant’s expectation for a normal interaction (Tronick et al. 1978; Lamb et al. 1987; Gusella et al. 1988; Weinberg and Tronick 1996; see Adamson and Frick 2003 for a review). Additionally, Tronick and colleagues (1978) also described a carry-over effect, with infants showing reduced engagement and a general decreased interaction in the interaction phase (i.e., the reunion) following the still-face episode.

Comparable effects have been recently reported in dogs: both laboratory (see Barrera et al. 2021) and pet dogs showed decreased proximity and contact-seeking behaviours during the still-face episode, along with increased solicitation behaviours such as begging (Cavalli et al. 2023), gaze aversion and vocalizations (Byrne et al. 2024) in the reunion phase.

However, the above-mentioned studies have primarily aggregated behaviours such as nose-licking, lip-wiping, yawning, and body shaking into cumulative stress scores (Byrne et al. 2024; Cavalli et al. 2023). Consequently, the potential communicative function of these specific visual signals (whose precise role is still under investigation, e.g., appeasement, greeting, or emotional expression) and how they might be employed to restore social interaction, remains less explored.

To investigate which communicative signals dogs employ to re-establish a positive social interaction we modified the classical version of the Still-Face Paradigm. Human social partners were physically separated from the dog by a large-meshed net with the aims of: (1) a better standardization of the dog-human interaction compared to a free physical interaction; (2) a focus on facial visual signals; (3) a future use of the paradigm to investigate visual communication in intraspecific context. The use of the net prevented dogs from relying on physical contact behaviours (e.g., pawing, pushing with the muzzle, licking the face of the human) to elicit the attention of the social partner, thereby targeting the investigation of dogs’ facial expressions. Importantly, a recent study on human-directed greeting interactions by Capitain & Wirosbki and colleagues (2025) showed that even with a net barrier between the animals and the social partners (i.e., their bonded human and a familiar human), dogs still exhibited displacement behaviours and changes in facial expressions, and kept interacting, indicating that their facial behavioural displays are not completely inhibited by the separation net.

It has been demonstrated that dogs are sensitive to people’s attentional state (Call et al. 2003; Schwab and Huber 2006) and change their behaviour accordingly (Gácsi et al. 2004; Kaminski et al. 2017). Also, previous research involving unsolvable tasks has demonstrated that dogs are sensitive to human attentional states and alternate their gaze less frequently when a human is facing away compared to when they are facing them (Marshall-Pescini et al. 2013). To investigate whether dogs change signals modalities (visual vs. auditory) according to human attentional state, we tested them in the classical **still-face** phase, in which the human sits in front to the dog, but remains unresponsive, and a novel **face-away** phase, in which the human is turned away, with their back facing the dog. Finally, dogs were tested with the owner and a familiar human partner (i.e., dog’s trainer, for a total of 6 different trainers) to investigate the role of social bonds in shaping dogs’ behavioural responses. Furthermore, the experimental design was structured so to include three interaction/reunion episodes, allowing us to assess potential boredom or fatigue in the subjects without having to rely on a control group exposed to a single, prolonged interaction phase.

This design enables us to expand previous findings about dogs’ attempts to repair disrupted interactions and whether they adapt their communicative strategies based on the perceived availability and attentiveness of the human. We predict that if specific behavioural displays (such as facial expressions, vocalizations, or gaze-seeking actions) serving a communicative function could also be used to restore social engagement, they will be exhibited more frequently during the non-interactive phases than during the interactive phase. In particular, we expect that dogs will modulate their signals according to human’s attentional state producing more **visual signals** when the human is looking at them (still-face) and more **auditory signals** when the human is not visually accessible (face-away).

Alternatively, following the social disruption of the still-face procedure, we might observe a significant decrease in these displays, consistent with withdrawal, frustration, or learned helplessness often seen in the Still-Face (Adamson and Frick 2003).

Finally, given the well-documented attachment bond between dogs and their owners (Topál et al. 1998; Prato-Previde et al. 2003), we predict stronger and more persistent efforts to restore the lost communication with their owner compared to the familiar human, reflecting the importance of the attachment figure in shaping dogs’ behaviour.

## Materials and methods

### Ethical statement

All the procedures were approved by the ethical committee of the University of Parma (approval numbers PROT.N.19/CESA/2023). The owners were informed about the experimental procedure and signed a consent form before the start of the experiment.

### Subjects

Thirty-seven domestic dogs of various breeds and of an age between 6 months and 11 years (x̄ 5,7 ± 3,8 years) were tested. The sample included 20 females (neutered = 12, intact = 8) and 17 males (neutered = 7, intact = 10) – see Supplementary Materials, Table S1 for detailed info. Subjects were recruited from the database of the University of Parma and from clients of the “Ca’Nina” Dog Training Centre (Desenzano del Garda, Italy).

### Experimental set-up

**Fig. 1:**
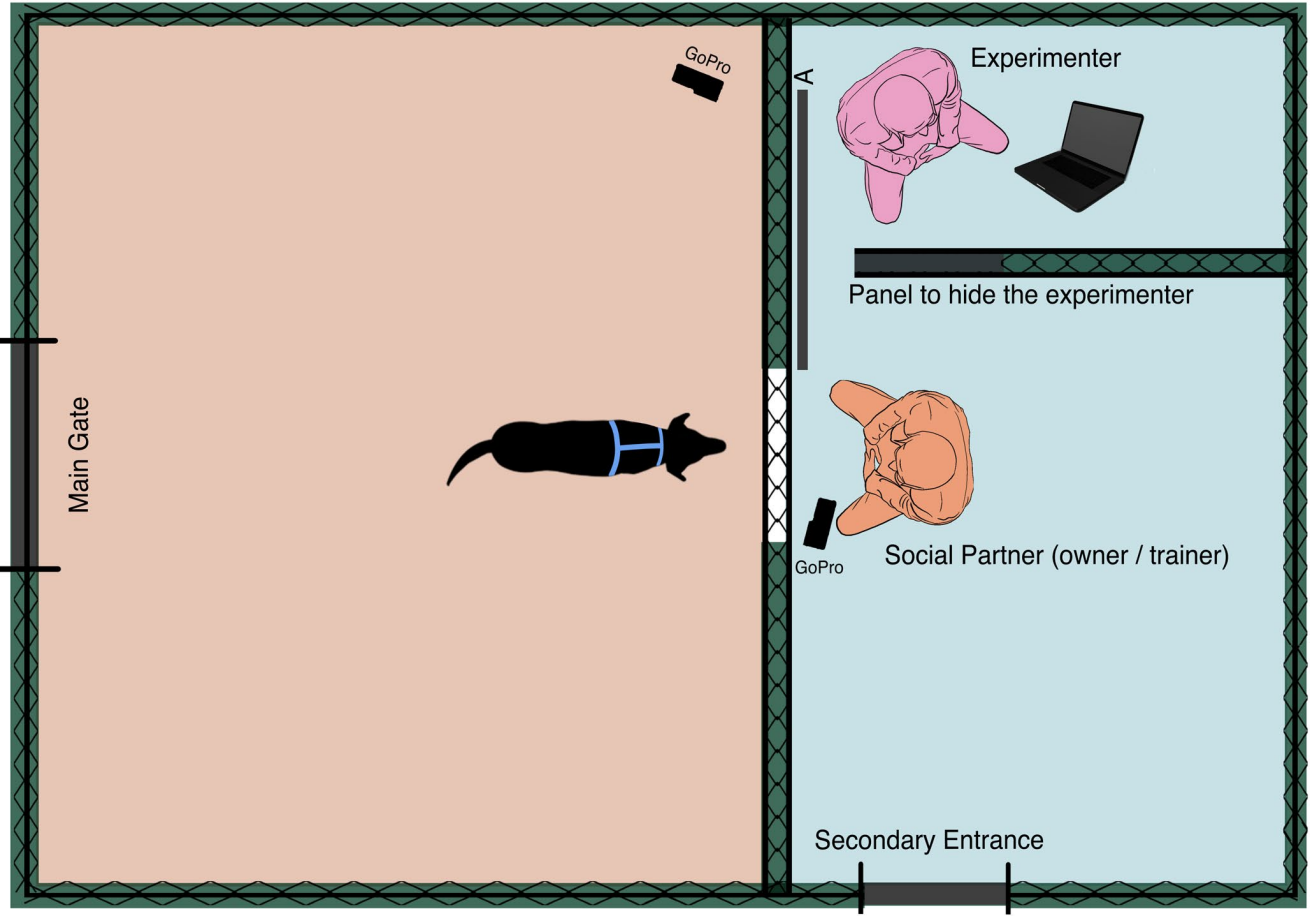
Graphical representation of the experimental areas (orange for the dog, blue for the people) and the position of the experimenter and the social partners during the different test phases. (A) represents the opaque panel that was opened and closed to distinguish the five episodes

The experiment was carried out in two fenced outdoor spaces located at the Ca’Nina Dog Training Centre and at the Department of Chemistry, Life Science and Environmental Sustainability in Parma, from February 2024 to June 2024. Both areas were of similar dimensions (approximately 5 m x 5 m), characterized by a larger section for the tested dog (Fig. 1, orange), a smaller one for the social partners (Fig. 1, blue). The areas were separated by a large-meshed net, with an opaque Plexiglas panel (Fig. 1, letter “A”) marking the transition between the consecutive episodes. They were enclosed by 1.8-m-high fences and isolated from the outside environment by dark sheets covering the entire perimeter. Two cameras (GoPro Hero10, Woodman Labs, San Mateo, CA, USA) were used for video recordings (placed as depicted in Fig. 1).

### Experimental procedure

Each dog was exposed consecutively to two conditions: owner experimental condition and familiar human experimental condition. To control for order effects, both the order of the social partners (Owner vs. Familiar Human) and the order of the non-interactive episodes (Still-Face vs. Face-Away) were counterbalanced across subjects (see Supplementary Materials, Table S1 for the detailed counterbalance list). To guarantee a consistent level of familiarity across subjects, the “familiar human” was selected according to specific inclusion criteria. The familiar humans included were dog trainers with whom the tested dogs had a history of regular direct interaction lasting for a minimum of 3 months, with a frequency of at least twice a month. The trainers’ role was not limited to instructing the caregivers; they actively engaged with the dogs in socialization tasks, walks, and playful, or sporting activities. This criterion ensured that the familiar human was a known individual with whom the dog had established a positive direct relationship.

The experimental conditions lasted less than 3 min (170s) and were divided as follow:

**Table 1:**
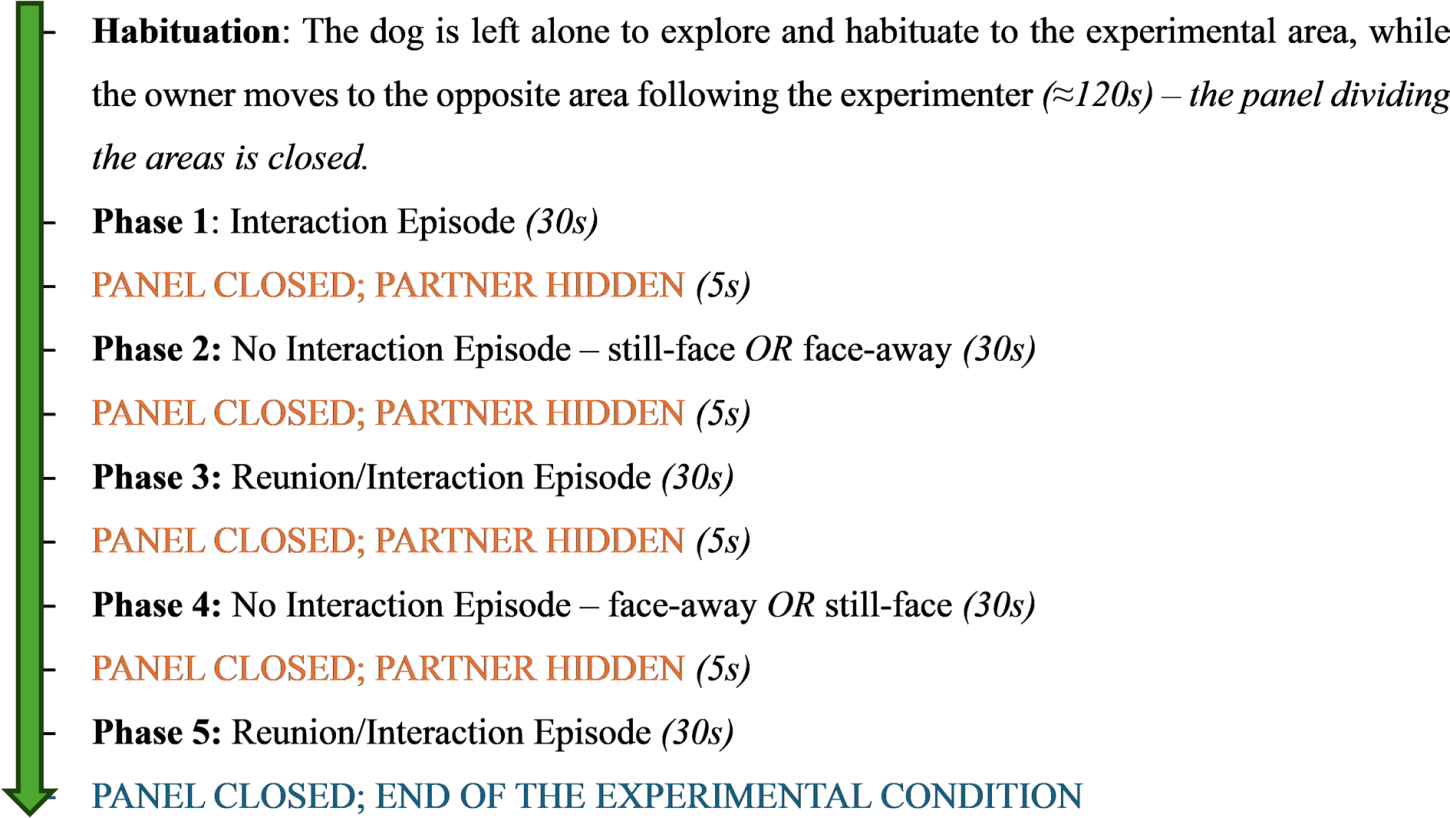
Timeline summarizing the test procedure, from initial habituation through all the experimental conditions

The second experimental condition with the other human partner followed the same scheme as the first, with the No Interaction phases (Still-Face and Face-Away) presented in the opposite order to the first time and without the two-minutes habituation period.

During the Interactions, human partners followed a standardized protocol to ensure consistent social stimulation. They were instructed to maintain a continuous stream of verbal communication using a positive, affiliative tone (e.g., calling the dog’s name, engaging in “small talk”) and to keep a friendly facial expression (smiling). Importantly, this verbal engagement was kept for the entire duration of the phase, regardless of the dog’s position, or attentional state.

### Behavioural coding

We developed an ethogram consisting of 40 behaviours (e.g., posture of the body, position of the dog, displacement behaviours, tail movements, vocalizations) and of 23 facial expressions and facial muscle movements redacted following DogFACS (Dog Facial Action Coding System – Waller et al. 2013). DogFACS is a coding system based on the Facial Action Coding System originally developed for humans (Ekman and Friesen 1978) and adapted to recognize and categorize detailed movements of the dog’s facial muscles and expressions.

Videos were coded using BORIS (Behavioral Observation Research Interactive Software, v7.13, Friad and Gamba 2016). Two experimenters (CC, CA) assessed inter-rater reliability using 25% of videos. Intra-class Correlations (ICC) were calculated using RStudio (ver. 2022.02.3) (R Core Team 2024), with a reliability ICC = 0.65–0.97 (values between 0.65 and 0.67 for tail postures and ear movements, above 0.70 for the remaining behaviours).

### Statistical analyses

Statistical analysis was performed using RStudio (ver. 2022.02.3), only behaviours expressed by more than 10% of dogs were included (Supplementary Materials Table 2).

Linear mixed models were used for the behaviours measured as “duration” (function “lmer”, package “lme4”, Bates et al. 2015). Behaviours measured as “frequency” were analysed using generalized linear mixed models with Poisson distributions (function “glmmTMB”, package “glmmTMB”, Brooks et al. 2017). A post-hoc sensitivity analysis (G*Power v. 3.1) indicated that with *N* = 37, α = 0.05, and Power = 0.80, the study was sensitive enough to detect small-to-medium effects (Cohen’s *f* = 0.21) in a repeated-measures framework.

We firstly ran a complete model including all phases (Int1 – Interaction 1, Int2 – Interaction 2, Int3 – Interaction 3, SF – Still-Face, FA – Face-Away), social partner ID (owner or familiar human) together with the following control predictors: testing location (Parma or Desenzano del Garda), order of the experimental condition (owner vs. familiar human), sex, and age. However, this model resulted unstable due to its statistical complexity and was therefore not considered further.

We then fitted a model including the interaction phases (Int1, Int2, Int3) and the social partner ID (owner or familiar human), to evaluate whether dogs’ behaviours varied across interaction phases. Dogs’ sex and age were entered as control predictors, while dog identity was included as a random effect. (see Table S2 and Supplementary Materials for detailed analyses and results).

We lastly ran a one-way interaction model between Phase (Int1, SF, FA) and Partner to test whether their combination significantly influenced behaviour. When the interaction was not significant- based on a comparison between the full and null models- the interaction term was removed, and the model was re-run to examine the main effects of Phase and Partner separately. Age and sex remained as control predictors, and dog ID was included as a random effect to account for individual variability.

To mantain 5% Type I error rate when testing the overall effect of the predictors on the response variable, we compared the Full model (including “Phase” – Int1, SF, FA – and “Partner” –owner, familiar human–), with a Null model that excludes them. This comparison was performed using a likelihood ratio test (Dobson 2002).

Post-hoc pairwise comparisons were then conducted using the “emmeans” function (package: “emmeans”, Lenth et al. 2020), which allows for a detailed exploration of the interactions between factors by performing pairwise comparisons within the levels of the other factor. Confidence intervals (CIs) for each pairwise comparison was also calculated (R base function “confint”).

Model diagnostic checks were performed: QQ-plots to assess residual normality; dispersion parameters to ensure that overdispersion was not an issue for count data; model fit using the diagnostics.plot() function from the “diagnostic_fcns.r” package to assess residuals and influential points.

The main analyses presented in this manuscript are based on the simplified model restricted to Int1, Still-Face, and Face-Away phases and their interaction with the social Partner (Owner vs. Familiar Human). To address our hypotheses, we examined dogs’ facial expressions and general behaviour across different phases of the procedure (first interaction, still-face, and face-away) and as a function of the social partner ID (Owner vs. Familiar Human). These analyses allowed us to focus on: (i) whether dogs displayed different behavioural patterns during the first interaction compared to the two non-interaction phases, (ii) whether differences emerged between the still-face and face-away episodes, and (iii) whether the behavioural response varied depending on whether the social partner was the owner or the familiar human.

## Results

The statistical model including the three interaction phases (Int1, Int2 and Int3) showed that the majority of behaviours were expressed more frequently or longer towards the owner than towards the familiar human. Behavioural intensity and frequency towards the familiar human were low and remained consistent across phases. In contrast, dogs displayed a markedly stronger behavioural response towards their owners during the first interaction, with substantially reduced intensity in the subsequent phases (Table S2 of Supplementary Materials for detailed contrasts).

Given that the most pronounced and informative behavioural patterns emerged during the first interaction phase, and that later phases showed attenuated and less variable responses, we focused our subsequent analyses on Int1 and the two non-interaction phases.

This analysis allowed us to focus on: (i) whether dogs displayed different behavioural patterns during the first interaction compared to the two non-interaction phases, (ii) whether differences emerged between the still-face and face-away episodes and (iii) whether the behavioural response varied depending on whether the social partner was the owner or the familiar human.

Overall, the results showed that dogs showed more facial expressions and visual displays, were more oriented and closer to the human partners during the first interaction phase (Int1) than during the non-interaction phases, (SF & FA). No differences were observed between the still-face and face-away phases suggesting a general decrease in social engagement after the first interaction. This final simplified model confirmed that several behaviours were expressed more frequently, or longer, towards the owner than towards the familiar human, reinforcing the idea that social bonding influences dogs’ communicative responses. Detailed outputs of the significant behaviours are provided in the Supplementary Materials and summarized in the Table 2 of the manuscript.

### Behaviours influenced both by specific phases and social partner

**Fig. 2:**
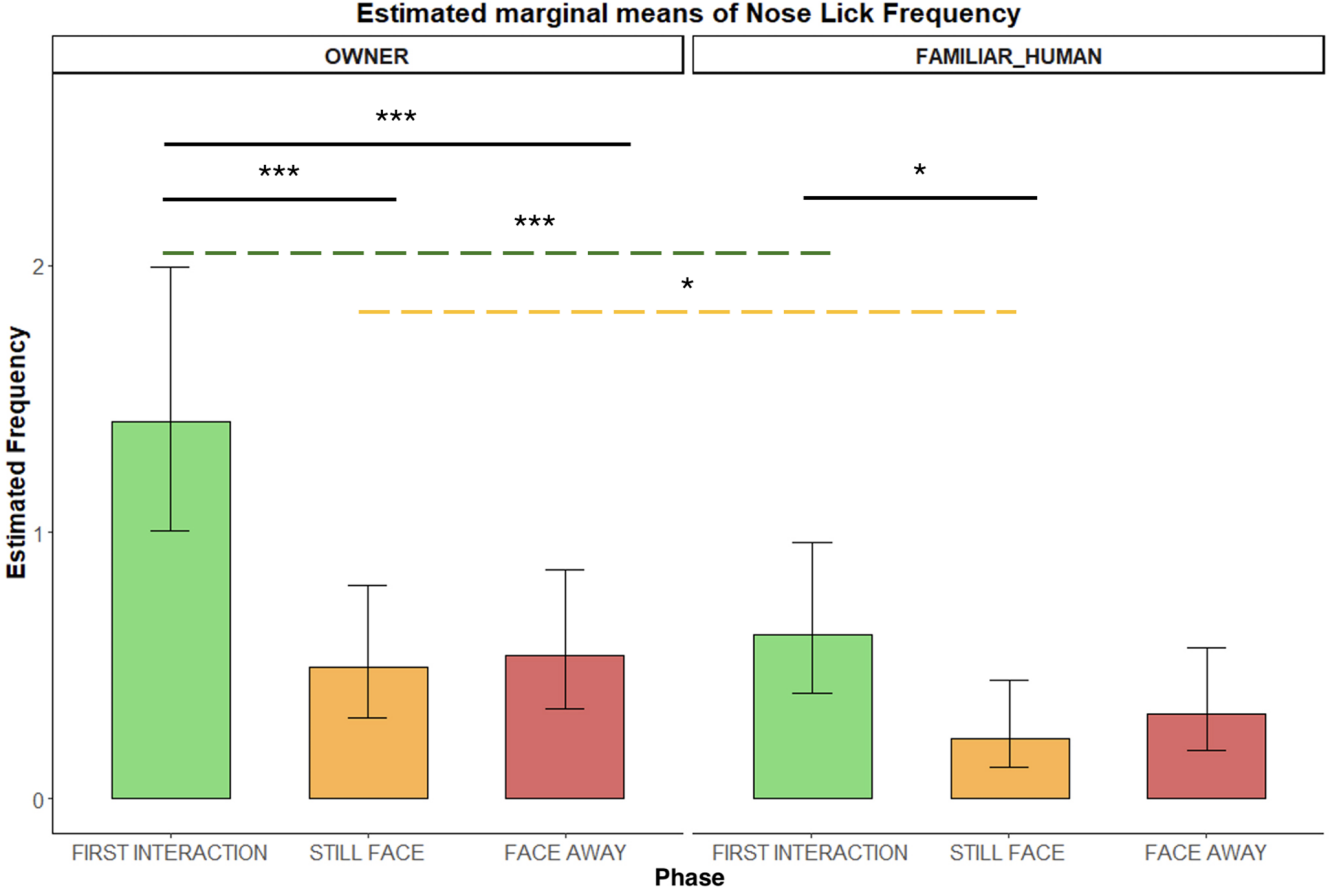
Frequency of nose-licking (AD137) during the Int1, SF, and FA phases with two social partners. Bar plots on the left show frequencies for the OWNER and those on the right for the FAMILIAR HUMAN. Significant p-values are highlighted above the barplots, black solid bars indicate phase differences, whereas the green and the yellow dashed bars indicate condition differences

*NOSE-LICKING (AD137)*. Nose-licking frequency was significantly higher with the OWNER compared to the FAMILIAR HUMAN, both during the first interaction *(p = 0.0003)* and the still-face *(p = 0.044).* Across phases with the owner, dogs displayed more nose-licking in the first interaction than in either still-face *(p = 0.0001)* or face-away *(p = 0.0002)* phases. A similar pattern emerged with the familiar human, with higher licking in the first interaction than in still-face (*p = 0.02* – Fig. 2).

**Fig. 3:**
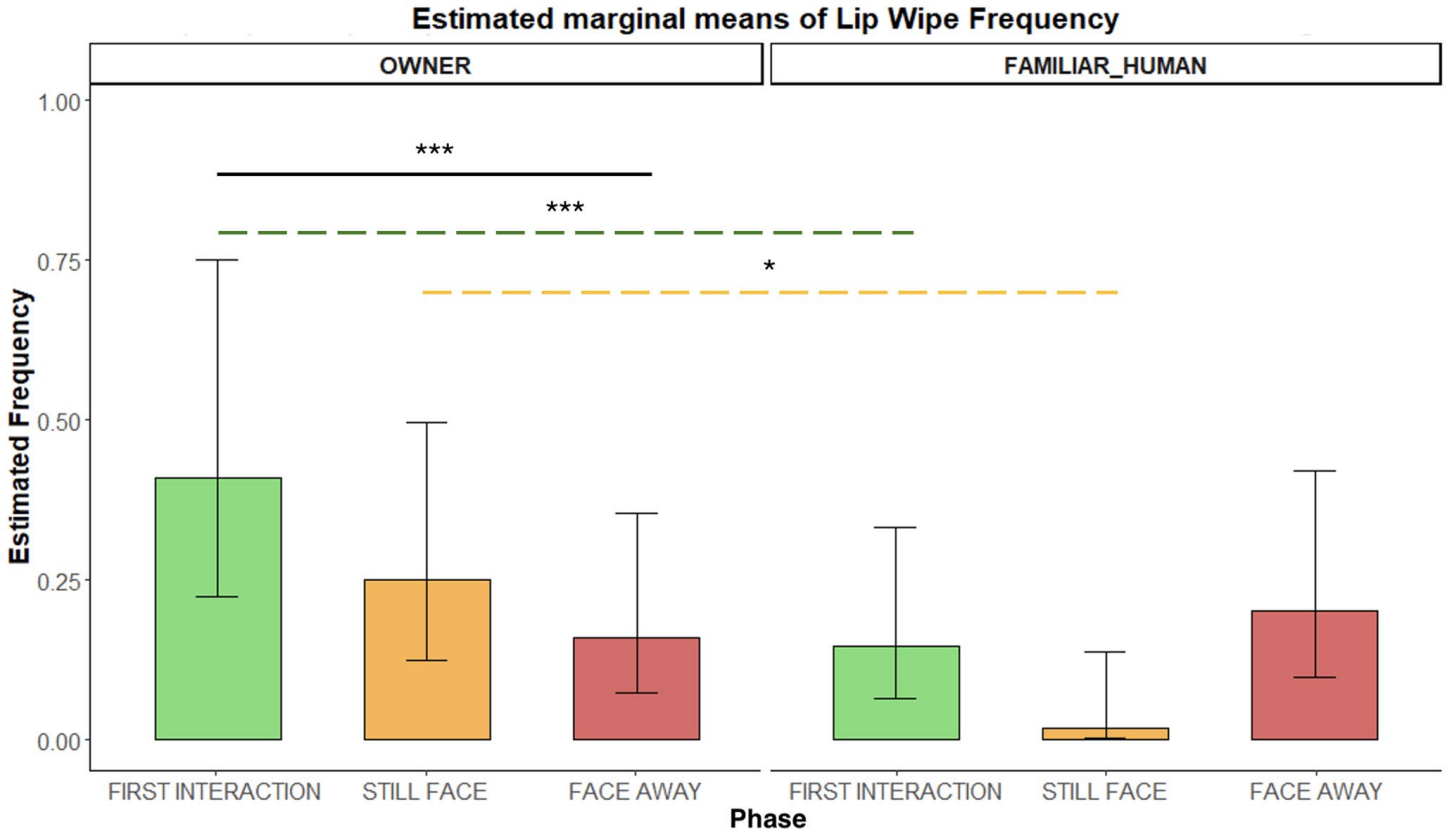
Frequency of lip-wiping (AD37) during the Int1, SF, and FA phases with two social partners. Bar plots on the left show frequencies for the OWNER and those on the right for the FAMILIAR HUMAN. Significant p-values are highlighted above the barplots, the black solid bar indicates phase differences, whereas the green and the yellow dashed bars indicate condition differences

*LIP-WIPING (AD37)*. Lip-wiping was expressed more frequently toward the OWNER than the FAMILIAR HUMAN during the first interaction *(p = 0.013)* and the still-face phase *(p = 0.012).* Across phases within the owner condition, dogs also performed more lip-wiping in the first interaction compared to the face-away phase (*p = 0.0001* – Fig. 3).

**Fig. 4:**
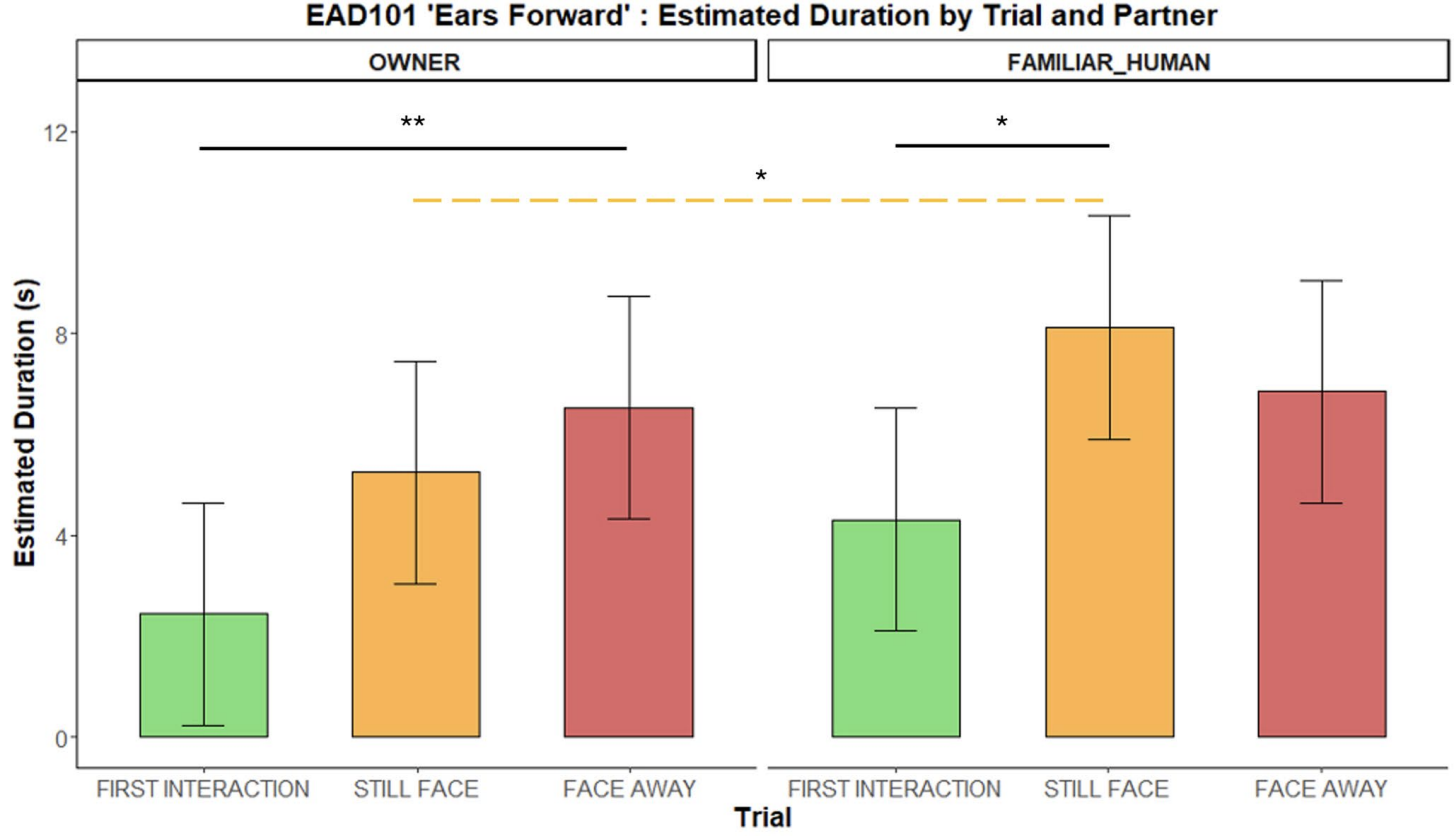
Duration of holding the ears forward (EAD101) during the Int1, SF, and FA phases with two social partners. Bar plots on the left show estimated durations for the OWNER and those on the right for the FAMILIAR HUMAN. Significant p-values are highlighted above the barplots, the black solid bars indicate phase differences, whereas the yellow dashed bar indicates SF differences across conditions

*EAR FORWARD (EAD101)*. Dogs held their ears forward more with the FAMILIAR HUMAN than the OWNER during the still-face phase *(p = 0.033)*. Across the phases, within the owner condition, dogs kept this ear position longer during the face-away phase compared to the first interaction *(p = 0.007).* In contrast, with the familiar human, dogs held their ears forward more during the still-face phase than in the first interaction (*p = 0.013* – Fig. 4).

**Fig. 5:**
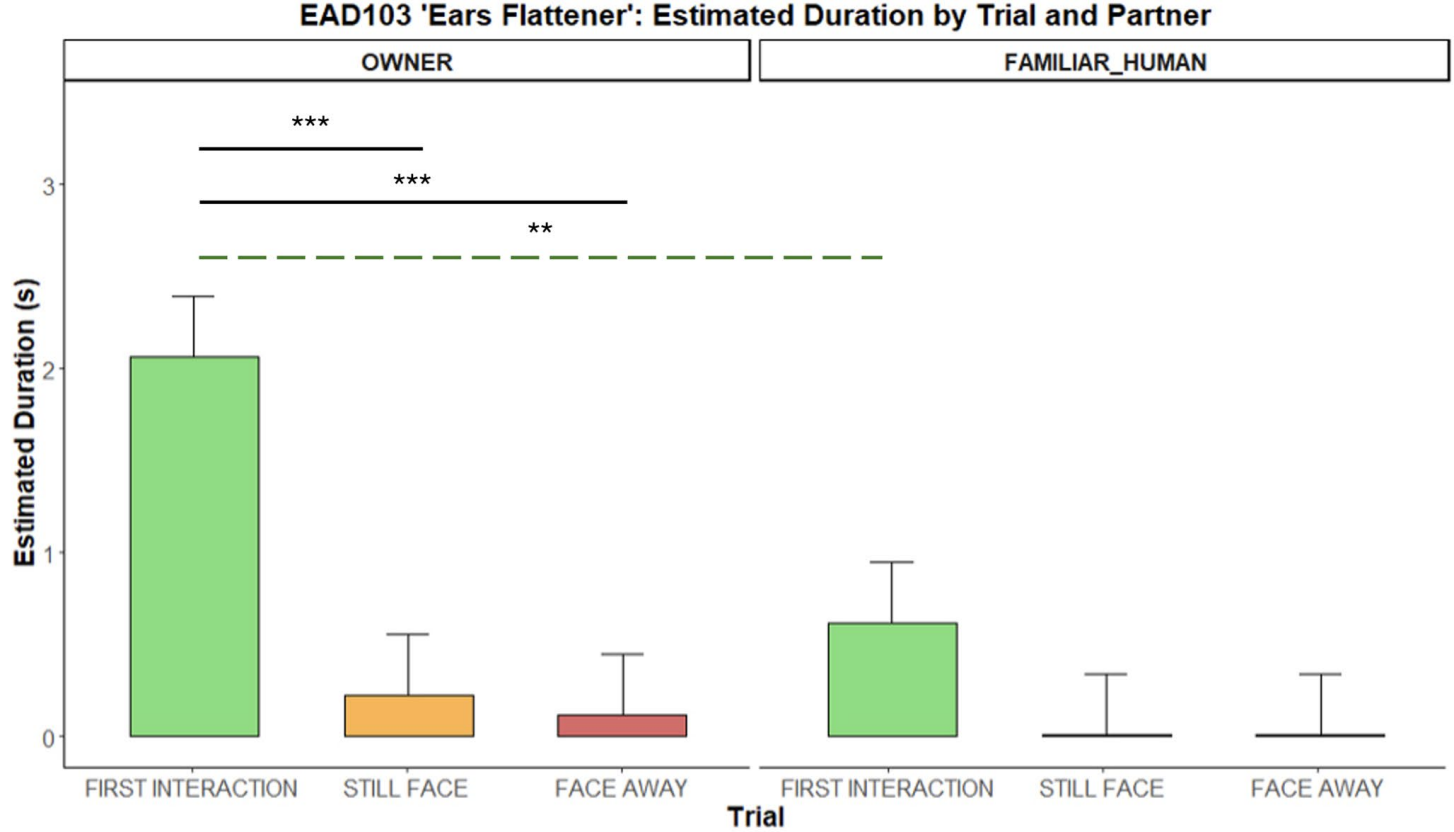
Duration of holding the ears flattened (EAD103) during the Int1, SF, and FA phases with two social partners. Bar plots on the left show estimated durations for the OWNER and those on the right for the FAMILIAR HUMAN. Significant p-values are highlighted above the barplots, the black solid bars indicate phase differences, whereas the green dashed bar indicates Int1 differences across conditions

*EAR FLATTENER (EAD103)*. Dogs held their ears flattener longer with the OWNER than the FAMILIAR HUMAN during the first interaction *(p = 0.002).* Across phases, within the owner condition, dogs kept the ears flattener longer in the first interaction than in either still-face *(p = 0.0002)* and face-away (*p = 0.0001)* phases (Fig. 5).

**Fig. 6:**
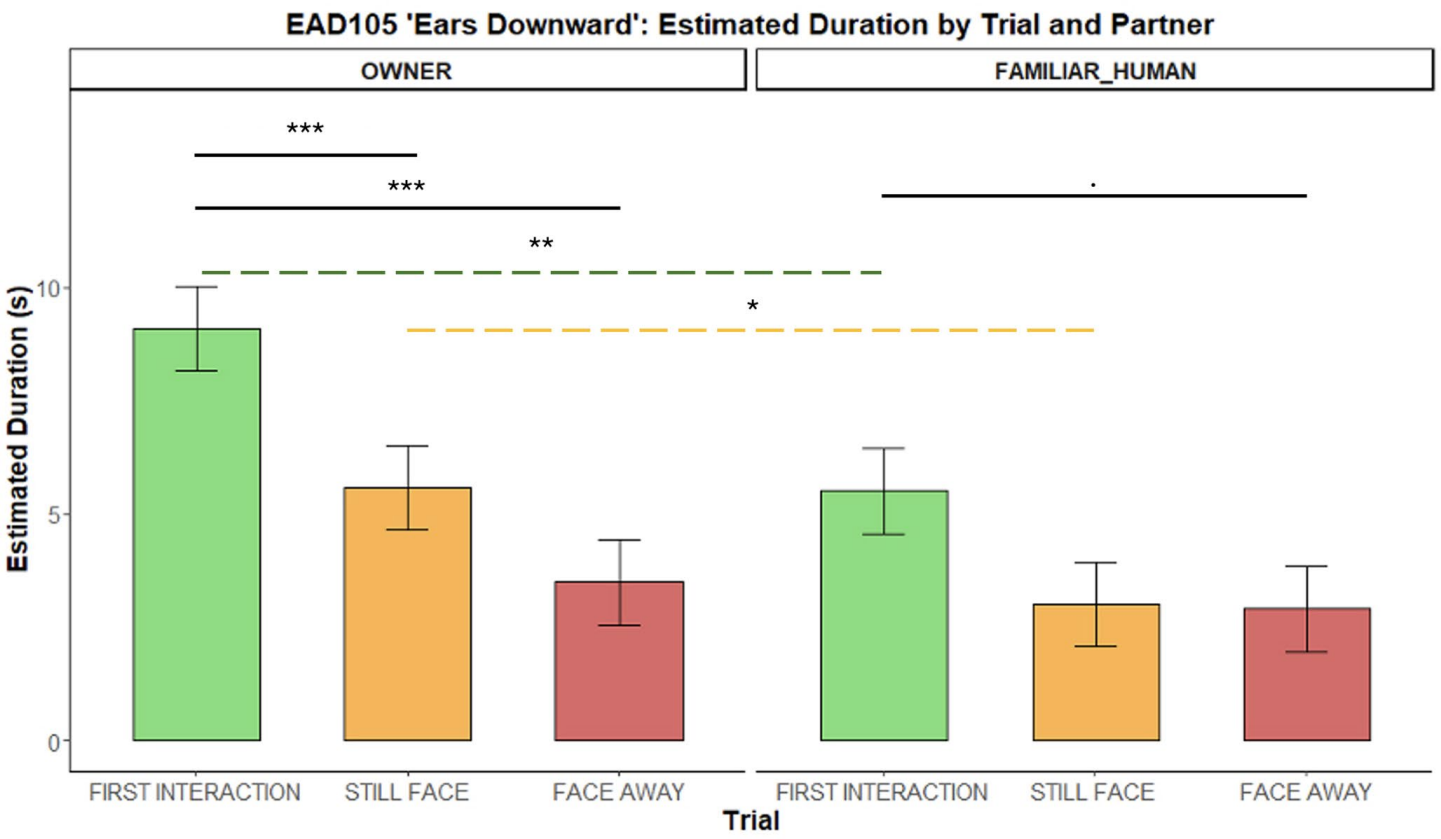
Duration of holding the ears downward (EAD105) during the Int1, SF, and FA phases with two social partners. Bar plots on the left show estimated durations for the OWNER and those on the right for the FAMILIAR HUMAN. Significant p-values are highlighted above the barplots, black solid bars indicate phase differences, whereas the green and the yellow dashed bars indicate respectively Int1 and SF differences across conditions

*EAR DOWNWARD (EAD105)*. Dogs held their ears downward longer with the OWNER than with the FAMILIAR HUMAN during the first interaction *(p = 0.002)* and the still-face phase *(p = 0.024).* Within the owner condition, dogs maintained this ear position longer in the first interaction compared to both the face-away *(p < 0.0001)* and still-face phases *(p = 0.006).* A similar trend was shown for the first interaction and face-away phases with the familiar human *(p = 0.057)* (Fig. 6).

*PROXIMITY: NEAR (< 1 m)*. Dogs stayed closer to the human partner when it was the owner, compared to the familiar human *(p = 0.008).* Within the owner condition, dogs stayed near longer during the first interaction compared to the face-away phase *(p = 0.02).*

**Fig. 7:**
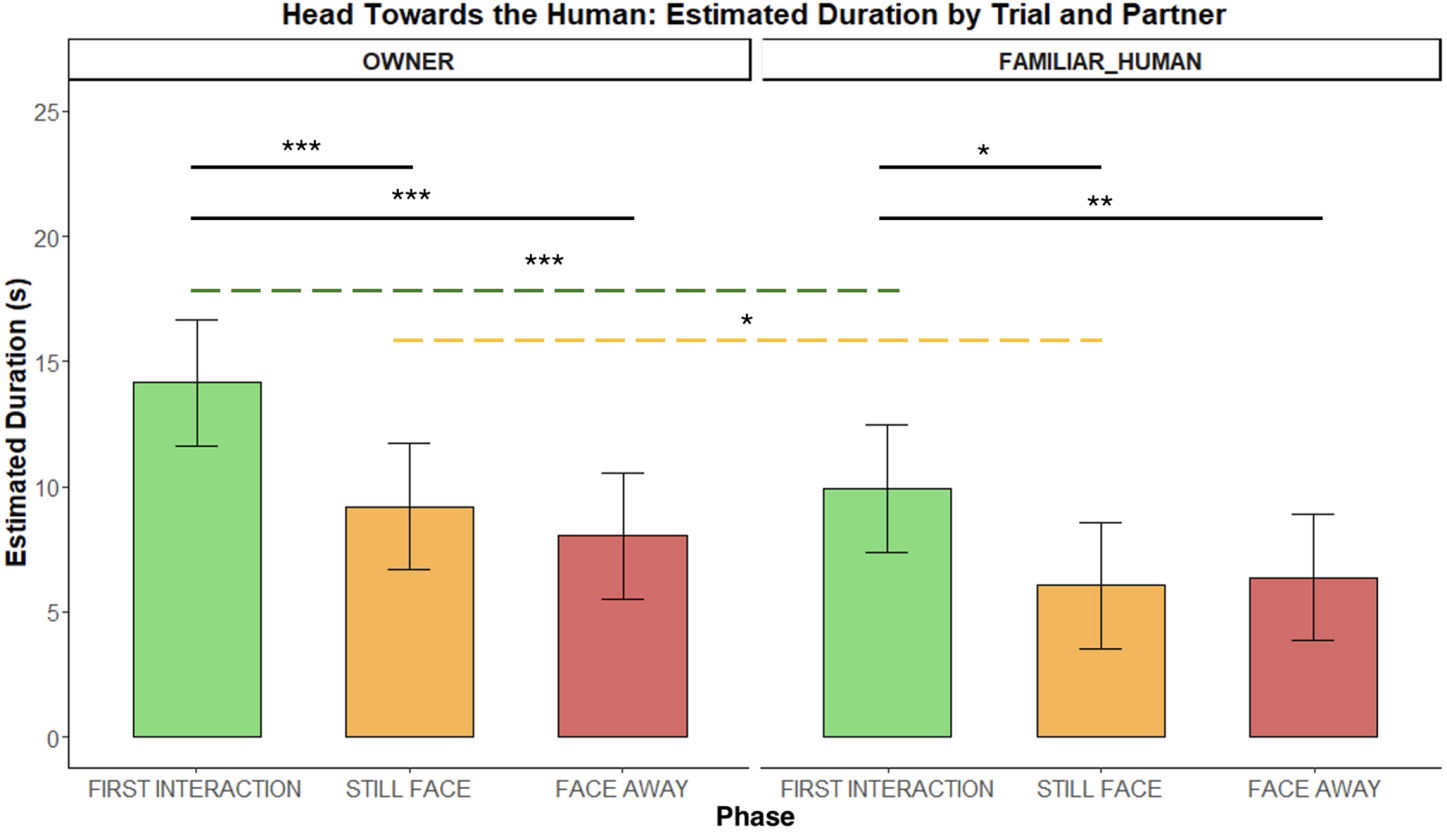
Duration of keeping the head towards the human during the Int1, SF, and FA phases with two social partners. Bar plots on the left show estimated durations for the OWNER and those on the right for the FAMILIAR HUMAN. Significant p-values are highlighted above the barplots, black solid bars indicate phase differences, whereas the green and the yellow dashed bars indicate respectively Int1 and SF differences across conditions

*HEAD HUMAN.* The social partner ID influenced this behaviour as dogs kept their head toward the human longer with the OWNER than the FAMILIAR HUMAN during the first interaction *(p = 0.0009)* and the still-face phase *(p = 0.013).* Across conditions this behaviour was most expressed during the first interaction compared to the two no-interaction phases both with the owner (Int1 vs. SF: *p* = 0.0003; Int1 vs. FA: *p* < 0.0001) and with the familiar human (Int1 vs. SF: *p* = 0.007; Int1 vs. FA: *p* = 0.014 – Fig. 7).

**Fig. 8:**
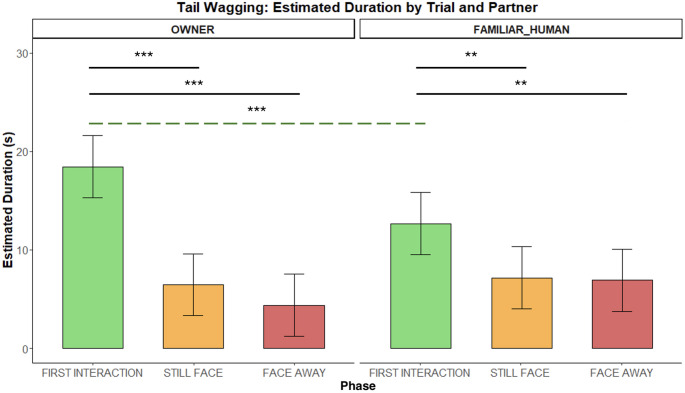
Duration of wagging the tail during the Int1, SF, and FA phases with two social partners. Bar plots on the left show estimated durations for the OWNER and those on the right for the FAMILIAR HUMAN. Significant p-values are highlighted above the barplots, the black solid bars indicate phase differences, whereas the green dashed bar indicates Int1 differences across conditions

*TAIL WAGGING.* Dogs wagged their tail longer with the OWNER than with the FAMILIAR HUMAN during the first interaction *(p = 0.001).* Tail wagging was also more expressed in the first interaction compared to the two non-interaction phases, both with the owner (Int1 vs. SF: *p* < 0.0001; Int1 vs. FA: *p* < 0.0001) and with the familiar human (Int1 vs. SF: *p* = 0.0043; Int1 vs. FA: *p* = 0.003 – Fig. 8).

### Behaviours influenced by specific phases

*HEAD TURNING (AD51 + AD52).* Dogs performed head-turning (both to the right and to the left) more frequently during the first interaction compared to the two non-interaction phases (Int1 vs. SF: *p* < 0.0001; Int1 vs. FA: *p* < 0.0001).

### Behaviours influenced by the social partner ID

*BLINKING (AU145)*. Dogs displayed eye blink more frequently when being tested by their OWNER compared to the FAMILIAR HUMAN (*p* = 0.0015), regardless of the phase.

*HEAD GATE*. Dogs kept their head towards the gate longer when they were tested by the FAMILIAR HUMAN compared to their OWNER (*p* = 0.0004) regardless of the phase.

**Table 2:**
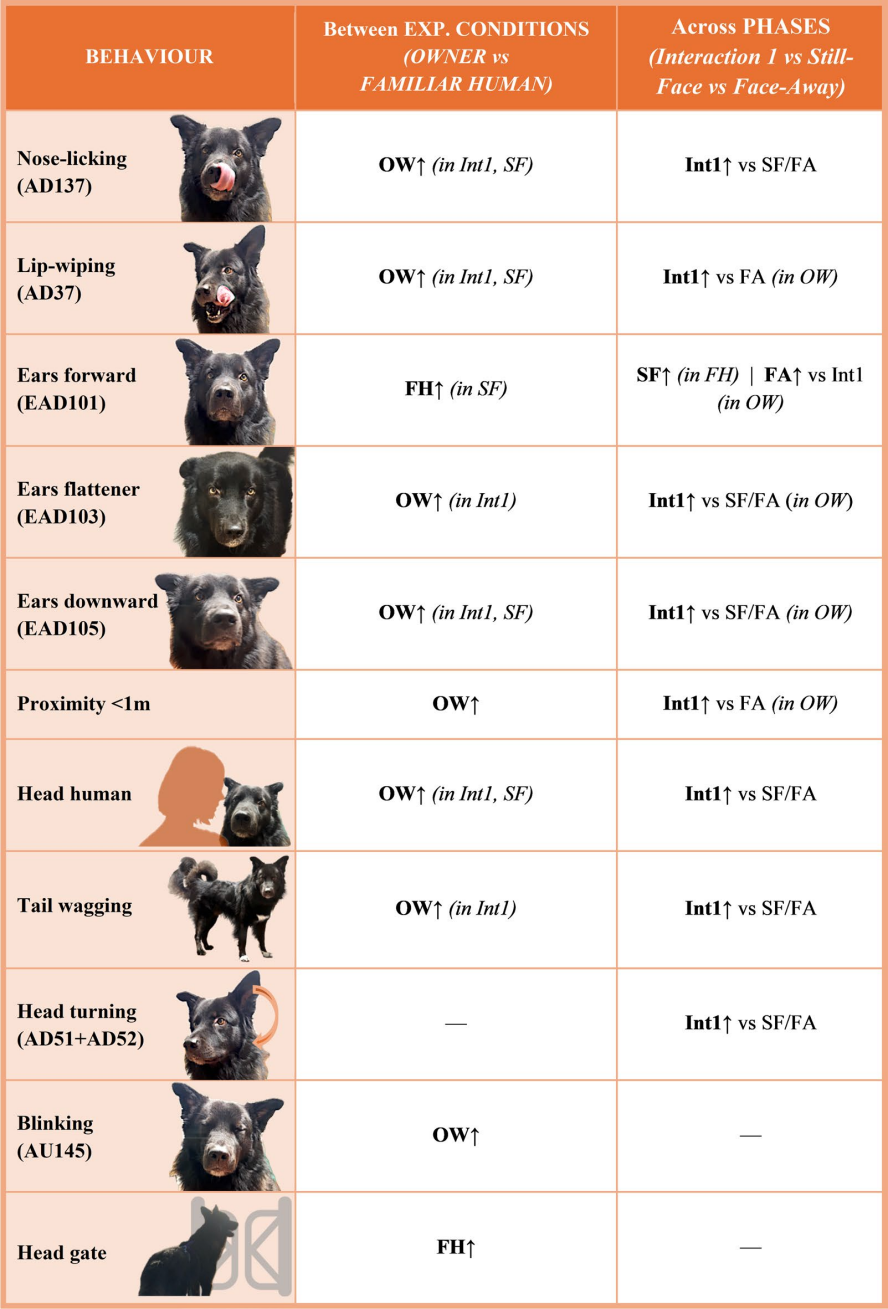
Summary of significant effects of social partner and phase on dogs’ behavioural displays

## Discussion

The principal aim of the present work was to investigate the still-face effect in domestic dogs hypothesizing that dogs: (1) would employ various communicative signals to restore social engagement with the human partner when interrupted; (2) would vary their strategies according to the attentional state of the human partner (“still-face” vs. “face-away” phases); (3) would show stronger persistence in these strategies toward their attachment figure (the owner) compared to a familiar human partner (their everyday trainer). Overall, dogs were most communicative during the first interaction (Int1), when the human partners were talking to them, and then decreased their efforts to engage with the human in the following non-interaction phases.

During Int1, dogs displayed a rich repertoire of visual and facial signals, including keeping their head and gaze oriented towards the human partner and ear and facial movements such as ear flattener and downward, lip/nose licking, eye-blinking and tail wagging. These results are in agreement with those recently published in dogs and wolf during a greeting test with bonded or familiar humans through a wire mesh (Capitain et al. 2025): wolves and dogs exhibited most of the pattern seen during the first interaction of our study as: gazing, tail wagging, nose lick, ears forward/flat/down. The expression of these behavioural patterns alongside closer proximity suggests that dogs were actively engaging in social communication even in the presence of a barrier. Other studies by Rehn and colleagues (2011 & 2013) found higher frequency of lip-wiping (i.e., lips licking in the papers) together with tail wagging upon the reunion episode with the owner/familiar person and suggest these are related to positive arousal or act as a reinstatement behaviour and a general response to the return of the person after a brief separation. The lip-wiping behaviour was also suggested to be an attempt to promote friendly and affiliative social interactions between attached individuals (Schenkel, 1967) and more recent studies (Pedretti et al. 2022, 2024) also suggested an effect of the audience and potentially communicative function of the lip-wiping and nose-licking displays previously interpreted as mere indicators of stress or negative affective states (Beerda et al. 1998, 2000; Horváth et al. 2008; Bremhorst et al. 2021). Other displays such as ear flattener or downward have been interpreted as communicative signals depending on the social context (Barrera et al. 2010, as “ears back and down”; Siniscalchi et al. 2018; Pedretti et al. 2022 – ear flattener; Pedretti et al. 2024 – ear downward). For instance, ear flattener increases in the presence of a human observer or when the human is attentive, suggesting a role in signalling emotional availability and facilitating affiliative interactions (Siniscalchi et al. 2018). Conversely, whilst the ear forward and adductor positions have been associated with attention, interest and anticipation (Pedretti et al. 2022; Bremhorst et al. 2019), the downward position (also “airplane ears” in the literature) might instead be associated with conflicting emotional states (Siniscalchi et al. 2018; Hecht and Horowitz^ 2015^). Finally, tail wagging behaviour and eye-blinking seem to be mostly related to social and affiliative contexts (tail wagging: Rehn et al. 2013; Siniscalchi et al. 2018; Ren et al. 2022; see Leonetti et al. 2024 for a comprehensive review; eye-blinking: Predetti et al. 2022; Koyasu et al. 2022). Particularly, the eye-blinking behaviour has recently gained attention in dog’s communicative scenario and was suggested to function as a social cue (Canori et al. 2025).

After the first interaction, the frequency and duration of all the behavioural patterns described above decreased significantly as expected on the basis of evidence of the “still-face” effect in both human infants and dogs. During the still-face phase, human infants show increased gaze aversion, less smiling and negative affect (see Adamson and Frick 2003 for a review) while dogs decreased gazing and contact (Barrera et al. 2021) and showed less proximity to the owner also during reunion episodes/final interaction (Cavalli et al. 2023; Byrne et al. 2024). Contrary to our initial predictions, the “face-away” phase did not elicit more auditory signals than the “still-face” one, and dogs behaved very similarly in the face-away and still-face phases. Our results are consistent with findings from a previous study (Marshall-Pescini et al. 2013) in which was observed that dogs did not increase physical contact (potentially to get attention) when the human was turned away. This suggests that the absence of increased signalling in the face-away condition is not unexpected given the dogs’ sensitivity to the audience’s attentional focus.

Another possible explanation for the lack of specific behaviours during these two phases is that the mesh barrier limited the dogs’ use of physical solicitation and begging behaviours showed by dogs during the still-face phase in Cavalli and colleagues’ study (2023).

Byrne and colleagues (2024) recently demonstrated that physical contact plays a crucial role in the still-face phenomenon, suggesting that the inability to physically touch the partner might have dampened the dogs’ persistence in seeking interaction. Furthermore, once the human partner became unresponsive, dogs appeared to quickly lose motivation to restart the interaction. While this decrease in behavioural intensity across phases could theoretically be attributed to fatigue or boredom, the relatively short duration of the test suggests a “carry-over effect” of the social disruption, similar to what has been observed in human infants (Tronick et al. 1978).

Similarly, the dogs in our study may have experienced a lasting impact of the interruption, consistent with a withdrawal response, or even learned helplessness, rather than simple habituation.

A consistent finding of the present study was that all the above-mentioned behavioural displays and use of facial expressions was mostly directed toward the owner compared to the familiar human. This aligns with our predictions and the attachment theory framework of dog–human relationships (Topál et al. 1998; Prato-Previde et al. 2003), reinforcing the idea that dogs perceive their owner as a secure base. In fact, when the experimental condition involved the owner, dogs showed greater proximity towards them compared to the familiar person, even when they did not reciprocate during the still-face/face-away episodes. Consistently with the “safe haven” effect described in the attachment theory (see Gácsi et al. 2013) such a pattern may reflect differences in the underlying social motivation: interactions with the attachment figure are intrinsically rewarding and help coping with potentially stressing situations like the present one and are therefore showed also during the interruptions when the owners were not responsive. On the other hand, interactions with a familiar partner do not appear to have the effect of emotional support, with dogs dismissing and keeping a greater distance from the human.

The stronger expression of the downward and flattener ear positions, eye-blinking and tail wagging towards the owner than towards the familiar human during the first interaction likely reflects higher social engagement and reinforce the view that these displays serve communicative functions in affiliative contexts. Capitain & Wirosbki and colleagues (2025), also reported that ear flattener was mostly shown by the animals during the greeting with their bonded human, altogether these results support that such positions may reflect a stronger motivation to communicate with the primary attachment figure.

## Conclusions

Our findings extend the evidence for a still-face effect in dogs and highlight the central role of the attachment bond in shaping communicative behaviours. The modulation of the behaviours accordingly to the human partner identity further highlights the importance of considering social bond as a crucial factor in experimental paradigms involving human–dog communication.

Future studies should test whether similar behavioural patterns are shown in intraspecific context and comparing the attachment figures with a completely unfamiliar human could further clarify whether the behaviours observed with the owner in this study were driven by attachment per se, or by the level of familiarity.

## Supplementary Information

Below is the link to the electronic supplementary material.

Supplementary Material 1

Supplementary Material 2

## Data Availability

All data on the behaviours supporting the findings of the study are included in the manuscript and the Supplementary Materials.

## References

[CR1] Adamson LB, Frick JE (2003) The Still Face: A History of a Shared Experimental Paradigm. Infancy 4(4):451–473. 10.1207/S15327078IN0404_01

[CR2] Barrera G, Guillén-Salazar F, Bentosela M (2021) Still-face effect in dogs (*Canis familiaris*): A pilot study. J Appl Anim Welfare Sci 24(4):364–377. 10.1080/10888705.2021.1923493 10.1080/10888705.2021.1923493 33988060

[CR3] Bates D, Mächler M, Bolker B, Walker S (2015) Fitting linear mixed-effects models using lme4. J Stat Softw 67(1). 10.18637/jss.v067.i01

[CR4] Beerda B, Schilder MBH, van Hooff JARAM, de Vries HW, Mol JA (1998) Behavioural, saliva cortisol and heart rate responses to different types of stimuli in dogs. Appl Anim Behav Sci 58(3–4):365–381. 10.1016/S0168-1591(97)00145-7

[CR5] Beerda B, Schilder MBH, van Hooff JARAM, de Vries HW, Mol JA (2000) Behavioural and hormonal indicators of enduring environmental stress in dogs. Anim Welf 9(1):49–62

[CR6] Bradshaw J, Rooney N (2016) Dog social behavior and communication. In: Serpell J (ed) The domestic dog: Its evolution, behavior and interactions with people, 2nd edn. Cambridge University Press, pp 133–159. 10.1017/9781139161800.008

[CR7] Bremhorst A, Sutter NA, Würbel H, Riemer S (2019) Differences in facial expressions during positive anticipation and frustration in dogs awaiting a reward. Sci Rep 9:19312. 10.1038/s41598-019-55714-6 31848389 10.1038/s41598-019-55714-6 PMC6917793

[CR8] Bremhorst A, Mills DS, Stolzlechner L, Würbel H, Riemer S (2021) Puppy dog eyes’ are associated with eye movements, not communication. Front Psychol 12:568935. 10.3389/fpsyg.2021.568935 33679505 10.3389/fpsyg.2021.568935 PMC7925631

[CR9] Brooks ME, Kristensen K, van Benthem KJ, Magnusson A, Berg CW, Nielsen A, Skaug HJ, Maechler M, Bolker BM (2017) glmmTMB balances speed and flexibility among packages for zero-inflated generalized linear mixed modeling. R J 9(2):378–400. 10.32614/RJ-2017-066

[CR10] Byrne M, Sawyer K, Johnston A (2024) Still face in pet dogs (Canis familiaris). J Comp Psychol 138(3):157–169. 10.1037/com0000371 38358709 10.1037/com0000371

[CR11] Caeiro C, Guo K, Mills D (2017) Dogs and humans respond to emotionally competent stimuli by producing different facial actions. Sci Rep 7, 15525. 10.1038/s41598-017-15091-4 10.1038/s41598-017-15091-4 PMC5686192 29138393

[CR12] Call J, Bräuer J, Kaminski J, Tomasello M (2003) Domestic dogs (Canis familiaris) are sensitive to the attentional state of humans. J Comp Psychol 117:257–263. 10.1037/0735-7036.117.3.257 14498801 10.1037/0735-7036.117.3.257

[CR13] Canori C, Travain T, Pedretti G, Fontani R, Valsecchi P (2025) If you blink at me, I’ll blink back. Domestic dogs’ feedback to conspecific visual cues. Royal Soc Open Sci 12(2):241703. 10.1098/rsos.241703 10.1098/rsos.241703 PMC11836432 39975663

[CR14] Capitain S, Wirobski G, Önsal Ç, Pedretti G, Bevilacqua V, Marshall-Pescini S, Range F (2025) Differences in dogs’ and wolves’ human-directed greeting behaviour: facial expressions, body language, and the problem of human biases. Anim Cogn 28(1):54. 10.1007/s10071-025-01978-7 40608143 10.1007/s10071-025-01978-7 PMC12226620

[CR15] Cavalli C, Dzik MV, Barrera G, Bentosela M (2023) Still-face effect in domestic dogs: Comparing untrained with trained and animal assisted interventions dogs. Learn Behav 51(4):428–445. 10.3758/s13420-023-00589-x 37407789 10.3758/s13420-023-00589-x

[CR43] R Core Team (2024) R: A language and environment for statistical computing [Computer software]. R Foundation for Statistical Computing. https://www.R-project.org/

[CR16] Dobson A (2002) An Introduction to Generalized Linear Models, 2nd Edition. Chapman& Hall CRC, USA

[CR17] Ekman P, Friesen WV (1978) Facial Action Coding System. Consulting Psychologist

[CR18] Friard O, Gamba M (2016) BORIS: A free, versatile open-source event‐logging software for video/audio coding and live observations. Methods Ecol Evol 7(11):1325–1330. 10.1111/2041-210x.12584

[CR20] Gácsi M, Miklósi Á, Varga O, Topál J, Csányi V (2004) Are readers of our face readers of our minds? Dogs (*Canis familiaris*) show situation-dependent recognition of human’s attention. Anim Cogn 7(3):144–153. 10.1007/s10071-003-0205-8 14669075 10.1007/s10071-003-0205-8

[CR19] Gácsi M, Maros K, Sernkvist S, Faragó T, Miklósi Á (2013) Human analogue safe haven effect of the owner: Behavioural and heart rate response to stressful social stimuli in dogs. PLoS ONE 8(3):e58475. 10.1371/journal.pone.0058475 23469283 10.1371/journal.pone.0058475 PMC3587610

[CR21] Gaunet F, Deputte BL (2011) Functionally referential and intentional communication in the domestic dog: Effects of spatial and social contexts. Anim Cogn 14(6):849–860. 10.1007/s10071-011-0418-1 21638003 10.1007/s10071-011-0418-1

[CR22] Gusella JL, Muir D, Tronick EZ (1988) The effect of manipulating maternal behavior during an interaction on three- and six-month-olds’ affect and attention. Child Dev 59(4):1111–1124. 10.2307/1130278 3168619 10.1111/j.1467-8624.1988.tb03264.x

[CR23] Haley DW, Stansbury K (2003) Infant stress and parent responsiveness: Regulation of physiology and behavior during still-face and reunion. Child Dev 74(5):1534–1546. 10.1111/1467-8624.00621 14552412 10.1111/1467-8624.00621

[CR24] Haley DW, Handmaker NS, Lowe J (2006) Infant stress reactivity and prenatal alcohol exposure. Alcoholism: Clin Experimental Res 30(12):2055–2064. 10.1111/j.1530-0277.2006.00251.x 10.1111/j.1530-0277.2006.00251.x 17117971

[CR25] Ham J, Tronick E (2006) Infant resilience to the stress of the still-face: Infant and maternal psychophysiology are related. Ann N Y Acad Sci 1094(1):297–302. 10.1196/annals.1376.038 17347365 10.1196/annals.1376.038

[CR26] Handelman B (2012) Canine behavior: a photo illustrated handbook. Woof and Word

[CR27] Hare B, Call J, Tomasello M (1998) Communication of food location between human and dog. Evol Communication 2:137–159. 10.1075/eoc.2.1.06har

[CR59] Hecht J, Horowitz A (2015) Seeing dogs: human preferences for dog physical attributes. Anthrozoös 28(1):153–163. 10.2752/089279315X14129350722217

[CR58] Horváth Z, Dóka A, Miklósi Á (2008) Affiliative and disciplinary behavior of human handlers during play with their dog affects cortisol concentrations in opposite directions. Horm Behav 54(1):107–114. 10.1016/j.yhbeh.2008.02.002 10.1016/j.yhbeh.2008.02.002 18353328

[CR28] Kaminski J, Hynds J, Morris P, Waller BM (2017) Human attention affects facial expressions in domestic dogs. Sci Rep 7(1):12914. 10.1038/s41598-017-12781-x 29051517 10.1038/s41598-017-12781-x PMC5648750

[CR60] Koyasu H, Goto R, Takagi S, Nagasawa M, Nakano T, Kikusui T (2022) Mutual synchronization of eyeblinks between dogs/cats and humans. Curr Zool 68(2):229–232. 10.1093/cz/zoab045 10.1093/cz/zoab045 PMC8962689 35355951

[CR30] Lamb M, Morrison D, Malkin C (1987) The development of infant social expectations in face-to-face interaction: A longitudinal study. Merrill-Palmer Q 33:241–254

[CR31] Leavens DA, Hopkins WD (1998) Intentional communication by chimpanzees: A cross-sectional study of the use of referential gestures. Dev Psychol 34(5):813–822. 10.1037/0012-1649.34.5.813 9779730 10.1037//0012-1649.34.5.813 PMC2080769

[CR32] Leavens DA, Hopkins WD, Bard KA (2005) Understanding the point of chimpanzee pointing: Epigenesis and ecological validity. Curr Dir Psychol Sci 14(4):185–189. 10.1371/journal.pone.0195182 18159225 10.1111/j.0963-7214.2005.00361.x PMC2151757

[CR33] Leaver SDA, Reimchen TE (2008) Behavioral responses of *Canis familiaris* to different tail lengths of a remotely-controlled life-sized dog replica. Behaviour 145:377–390

[CR34] Lenth R, Buerkner P, Herve M, Love J, Riebl H, Singmann H (2020) emmeans: Estimated marginal means, aka least-squares means (R package version 1.4.4) [Computer software]. CRAN. https://cran.r-project.org/web/packages/emmeans/index.html

[CR35] Leonetti S, Cimarelli G, Hersh TA, Ravignani A (2024) Why do dogs wag their tails? Biol Lett 20(1):20230407. 10.1098/rsbl.2023.0407 38229554 10.1098/rsbl.2023.0407 PMC10792393

[CR36] Marshall-Pescini S, Colombo E, Passalacqua C, Merola I, Prato-Previde E (2013) Gaze alternation in dogs and toddlers in an unsolvable task: evidence of an audience effect. Anim Cogn 16(6):933–943. 10.1007/s10071-013-0627-x 23543361 10.1007/s10071-013-0627-x

[CR37] Miklósi Á, Polgárdi R, Topál J, Csányi V (2000) Intentional behaviour in dog-human communication: An experimental analysis of showing behaviour in the dog. Anim Cogn 3(3):159–166. 10.1007/s100710000072

[CR57] Passalacqua C, Marshall-Pescini S, Barnard S, Lakatos G, Valsecchi P, Prato-Previde E (2011) Human-directed gazing behaviour in puppies and adult dogs (*Canis lupus familiaris*). Anim Behav 82(5):1043–1050. 10.1016/j.anbehav.2011.07.039

[CR40] Pedretti G, Canori C, Marshall-Pescini S, Palme R, Pelosi A, Valsecchi P (2022) Audience effect on domestic dogs’ behavioural displays and facial expressions. Sci Rep 12(1):9747. 10.1038/s41598-022-13566-7 35697913 10.1038/s41598-022-13566-7 PMC9192729

[CR38] Pedretti G, Canori C, Biffi E, Marshall-Pescini S, Valsecchi P (2023) Appeasement function of displacement behaviours? Dogs’ behavioural displays exhibited towards threatening and neutral humans. Anim Cogn 26(3):943–952. 10.1007/s10071-023-01742-9 36662320 10.1007/s10071-023-01742-9 PMC10066101

[CR39] Pedretti G, Canori C, Costantini E et al (2024) Intra and interspecific audience effect on domestic dogs’ behavioural displays and facial expressions. Sci Rep 14:9546. 10.1038/s41598-024-58757-6 38664496 10.1038/s41598-024-58757-6 PMC11045831

[CR41] Prato-Previde E, Marshall-Pescini S (2014) Social looking in the domestic dog. In A. Horowitz (Ed.), Domestic dog cognition and behavior: The scientific study of Canis familiaris (pp. 101–131). Springer. 10.1007/978-3-642-53994-7_5

[CR42] Prato-Previde E, Custance DM, Spiezio C, Sabatini F (2003) Is the dog-human relationship an attachment bond? An observational study using Ainsworth’s strange situation. Behaviour 140(2):225–254. 10.1163/156853903321671514

[CR45] Rehn T, McGowan RTS, Keeling LJ (2013) Evaluating the Strange Situation Procedure (SSP) to assess the bond between dogs and humans. PLoS ONE 8(2):e56938. 10.1371/journal.pone.0056938 23437277 10.1371/journal.pone.0056938 PMC3577677

[CR46] Ren W, Wei P, Yu S, Zhang YQ (2022) Left-right asymmetry and attractor-like dynamics of dog’s tail wagging during dog-human interactions. iScience 25(8):104747. 10.1016/j.isci.2022.104747 35942104 10.1016/j.isci.2022.104747 PMC9356099

[CR47] Schenkel R, Submission (1967) Its Features and Function in the Wolf and Dog, American Zoologist, 7(2):319–329. 10.1093/icb/7.2.319

[CR48] Schwab C, Huber L (2006) Obey or not obey? Dogs (*Canis familiaris*) behave differently in response to attentional states of their owners. J Comp Psychol 120(3):169–175. 10.1037/0735-7036.120.3.169 16893253 10.1037/0735-7036.120.3.169

[CR50] Siniscalchi M, Lusito R, Vallortigara G, Quaranta A (2013) Seeing Left- or Right-Asymmetric Tail Wagging Produces Different Emotional Responses in Dogs. Curr Biol 23(22):2279–2282. 10.1016/j.cub.2013.09.027 24184108 10.1016/j.cub.2013.09.027

[CR49] Siniscalchi M, d’Ingeo S, Minunno M, Quaranta A (2018) Communication in Dogs. Animals 8(8):131. 10.3390/ani8080131 30065156 10.3390/ani8080131 PMC6116041

[CR51] Topál J, Miklósi Á, Csányi V, Dóka A (1998) Attachment behavior in dogs (Canis familiaris): A new application of Ainsworth’s (1969) Strange Situation Test. J Comp Psychol 112(3):219–229. 10.1037/0735-7036.112.3.219 9770312 10.1037/0735-7036.112.3.219

[CR52] Tronick EZ, Als H, Adamson L, Wise S, Brazelton TB (1978) The infant’s response to entrapment between contradictory messages in face-to-face interaction. J Am Acad Child Psychiatry 17(1):1–13. 10.1016/S0002-7138(09)62273-1 632477 10.1016/s0002-7138(09)62273-1

[CR54] Van der Borg JAM, Schilder MBH, Vinke CM, de Vries H (2015) Dominance in Domestic Dogs: A Quantitative Analysis of Its Behavioural Measures. PLoS ONE 10(8):e0133978. 10.1371/journal.pone.0133978 26309101 10.1371/journal.pone.0133978 PMC4556277

[CR55] Waller B, Peirce K, Correia-Caeiro C, Oña L, Burrows A, Mccune S, Kaminski J (2013) Paedomorphic Facial Expressions Give Dogs a Selective Advantage. PLoS ONE 8:e82686. 10.1371/journal.pone.0082686 24386109 10.1371/journal.pone.0082686 PMC3873274

[CR56] Weinberg MK, Tronick EZ (1996) Infant affective reactions to the resumption of maternal interaction after the still-face. Child Dev 67(3):905–914. 10.2307/1131869 8706534

