# Supplementary material for: Can you read my poker-face? Adapting the still-face paradigm to explore dog’s interspecific communication
Source: Anim Cogn. 2026 Mar 14;29(1):34. doi: 10.1007/s10071-026-02059-z (PMC13002743; doi:10.1007/s10071-026-02059-z)
Supplement: Supplementary file 2 — Supplementary Material 2 [file 10071_2026_2059_MOESM2_ESM.docx]

**Supplementary Material**

The following supplementary information and tables referrer to:
“*Can you read my poker face? Adapting the Still-Face Paradigm to explore dog’s interspecific communication*”

***Table 1.*** Subjects involved in the study & order of the human partner and no-interaction phases

| subject | age | sex | neutered | breed | breed_type | human_order | location | familiar_human |
| --- | --- | --- | --- | --- | --- | --- | --- | --- |
| Ska | 10 | F | yes | terrier_mix | mix | FH_OW | Parma | Paola |
| Lea | 12 | F | yes | hunter_mix | mix | OW_FH | Parma | Barbara |
| Cleo | 9,5 | F | yes | border_collie | shepherd | OW_FH | Parma | Chiara |
| Alaska | 6 | M | no | lupino_del_gigante | shepherd | FH_OW | Parma | Chiara |
| Paco | 10 | M | yes | weimaraner | hunter | FH_OW | Parma | Chiara |
| Oscar | 7 | M | yes | mix | mix | OW_FH | Parma | Giulia |
| Quickly | 10 | M | yes | border_collie | shepherd | FH_OW | Parma | Barbara |
| Ziva | 9 | F | yes | weimaraner | hunter | OW_FH | Parma | Chiara |
| Olivia | 3,5 | F | no | rhodesian | hunter | FH_OW | Parma | Chiara |
| Kappa | 1 | M | no | short_hair_pointer_mix | hunter | OW_FH | Parma | Chiara |
| Luna | 9 | F | yes | retriever_mix | hunter | FH_OW | Parma | Barbara |
| Buddy | 9 | M | yes | retriever_mix | hunter | OW_FH | Parma | Barbara |
| Macchia | 11 | M | yes | shepherd_mix | mix | OW_FH | Parma | Barbara |
| Spritz | 2 | M | no | shepherd_mix | shepherd | OW_FH | Parma | Paola |
| Vilu | 10 | F | no | mix | mix | OW_FH | Parma | Paola |
| Mia | 8 | F | yes | cocker_mix | hunter | FH_OW | Parma | Paola |
| Tuono | 8 | M | yes | mix | mix | FH_OW | Parma | Paola |
| Cody |  | M | no | jack_russell | hunter | FH_OW | Parma | Paola |
| Margie |  | F | yes | mix_yorkie | mix | FH_OW | Parma | Paola |
| Navarre | 2 | M | no | border_mix | shepherd | OW_FH | Desenzano | Giulia |
| Alice | 4 | F | no | oropa_mix | shepherd | OW_FH | Desenzano | Sara |
| Sally | 1 | F | no | golden_retriever | hunter | FH_OW | Desenzano | Laura |
| Balto | 3 | M | no | border_mix | shepherd | OW_FH | Desenzano | Laura |
| Uma | 11 | F | yes | fonnese_mix | shepherd | OW_FH | Desenzano | Laura |
| Mia | 3,5 | F | yes | siberian_husky | primitive | OW_FH | Desenzano | Laura |
| Mako | 1 | F | no | labrador_retriever | hunter | OW_FH | Desenzano | Laura |
| Dylan_Bull | 7 | M | yes | amstaff | bull | FH_OW | Desenzano | Laura |
| Dana | 2 | F | no | malinois_x_czech_wd | shepherd | FH_OW | Desenzano | Laura |
| Dea | 2 | F | yes | belgian_malinois | shepherd | FH_OW | Desenzano | Laura |
| Rio | 1 | M | no | amstaff | bull | OW_FH | Desenzano | Sara |
| Lilli | 0,5 | F | no | cocker_spaniel | hunter | OW_FH | Desenzano | Sara |
| Zara | 1 | F | no | cocker_spaniel | hunter | FH_OW | Desenzano | Sara |
| Balì | 0,5 | M | no | mix_golden_gsd | hunterxshepherd | FH_OW | Desenzano | Sara |
| Dylan | 4 | M | no | mix_hunter | mix | OW_FH | Desenzano | Giulia |
| Lou | 7 | F | yes | mix_cocker | hunter | OW_FH | Desenzano | Giulia |
| Wendy | 7 | F | yes | husky_mix | mix | FH_OW | Desenzano | Giulia |
| Ares | 7 | M | no | dobermann | hunter | FH_OW | Desenzano | Chiara |

| OW_FH | StillFace/FaceAway_OWNER | FaceAway/StillFace_FAMILIAR |
| --- | --- | --- |
| OW_FH | FaceAway/StillFace_OWNER | StillFace/FaceAway_FAMILIAR |
| FH_OW | StillFace/FaceAway_FAMILIAR | FaceAway/StillFace_OWNER |
| FH_OW | FaceAway/StillFace_FAMILIAR | StillFace/FaceAway_OWNER |

***Complete ethograms of the study divided into two different categories: DogFACS ethogram (based on Dog Facial Action Coding System manual) and general behaviours ethogram.***

***Table 2.*** Complete ethogram of all the behaviours coded.

|  | **Variables** | **Coded & analysed as:** |
| --- | --- | --- |
| **DogFACS** | Blinking (AU145) | Frequency |
|  | Inner Brow Raiser (AU101) | Duration |
|  | Nose-Licking (AD137) | Frequency |
|  | Lip-Wiping (AD37) | Frequency |
|  | Head Turning (AD51&52) | Frequency |
|  | Ears Forward (EAD101) | Duration |
|  | Ears Adductor (EAD102) | Duration |
|  | Ears Flattener (EAD103) | Duration |
|  | Ears Rotator (EAD104) | Duration |
|  | Ears Downward (EAD105) | Duration |
|  | Panting (AD126) | Duration |
|  | Sniffing the Social Partner (AD40) | Duration |
|  | Sniffing Around (AD40-A) | Duration |
|  | Head Tilt Left (AD55) | Duration |
|  | Head Tilt Right (AD56) | Duration |
|  | Chewing (AD81) | Duration |
|  | Body Shake (AD160) | Frequency |
|  | Mouth Stretch (AU27) | Frequency |
|  | Nose Wrinkler + Upper Lip Raiser (AU109+110) | Duration |
|  | Eye Closure (AU143) | Duration |
|  | Blow (AD33) | Duration |
|  | Softening the Eyes | Duration |
| **General** **Behaviours** | Proximity: NEAR (<1mt) | Duration |
|  | Proximity: FAR (>1mt) | Duration |
|  | Vocalizations  (Barking + Whining + Growling) | Duration |
|  | Head Elsewhere | Duration |
|  | Head Social Partner | Duration |
|  | Head Gate | Duration |
|  | Tail Wagging | Duration |
|  | Laying Down | Duration |
|  | Sitting | Duration |
|  | Standing | Duration |
|  | Eating Grass | Duration |
|  | Gasping | Duration |
|  | Self-Scratching | Frequency |
|  | Stretching | Frequency |
|  | Sneezing | Frequency |
|  | Marking | Frequency |
|  | Arching the Back | Frequency |
|  | Freezing | Duration |
|  | High Posture | Duration |
|  | Low Posture | Duration |
|  | Neutral Posture | Duration |
|  | Locomotion states (Trotting / Walking / Still / Back) | Duration |
|  | Digging | Duration |
|  | Pointing | Duration |
|  | Paw Lifting | Frequency & Duration |
|  | Paw Tapping | Frequency & Duration |
|  | Trembling | Duration |
|  | Resting | Duration |

***Detailed results of the models including all the phases (Int1, Int2, Int3, SF, FA) and control predictors (Social Partner, Sex, Age, Neutered State, Human Order, Location)***

1. ***Behaviours influenced both by specific Phases and Social Partner***

NOSE-LICKING (AD137). Results of the GLMM and the full-null model comparison revealed a significant influence of the factors “Partner” and “Phase” on the nose-licking (full–null model comparison: x^2^ = 61.666; df = 9; p < 0.001). Dogs displayed nose lick more frequently when being tested by their owner compared to the familiar human (PartnerOWNER: Es. 0.619 ± 0.129, p-value < 0.001, 95% CI [0.36, 0.87] – Figure 4), during the first interaction compared to the non-interaction episodes and the final interaction (Int1-SF: Es. 1.034 ± 0.202, p-value < 0.0001, 95% CI [0.48, 1.16]; FA-Int1: Es. -0.862 ± 0.190, p-value = 0.0001, 95% CI [-1.38, -0.34]; Int1-Int3: Es. 0.629 ± 0.175, p-value = 0.003, 95% CI [0.15, 1.11]) and during the second interaction compared to the still-face episode (Int2-SF: Es. 0.595 ± 0.216, p-value = 0.047, 95% CI [0.005, 1.18]).

EARS FORWARD (EAD101). The duration of the ears forward behaviour was influenced by the factors “Partner” and “Phase” (full–null model comparison: x^2^ = 32.31; df = 9; p = 0.0002). Dogs kept the ears forward when the familiar human was testing them (PartnerOWNER: Es. -1.260 ± 0.573, p-value = 0.029, 95% CI [-2.39, -0.13]) (Figure 8). Furthermore, this position was kept for longer during the two no-interaction episodes compared to the first and the third interaction (FA-Int1: Es. 3.314 ± 0.89, p-value = 0.002, 95% CI [0.86, 5.77]; FA-Int3: Es. 2.984 ± 0.89, p-value = 0.008, 95% CI [0.53, 5.44]; Int1-SF: Es. -3.311 ± 0.89, p-value = 0.002, 95% CI [-5.77, -0.85]; Int3-SF: Es. -2.981 ± 0.89, p-value = 0.002, 95% CI [-5.44, -0.53]).

EARS FLATTENER (EAD103). The duration of the ears flattener behaviour was influenced by the factors “Partner” and “Phase” (full–null model comparison: x^2^ = 32.83; df = 9; p = 0.0001). Specifically, the testing phase influenced the time dogs kept their ears flattened, resulting in a longer time this behaviour was expressed during the first interaction compared to all the remaining phases (Int1-SF: Es. 1.224 ± 0.278, p-value = 0.0001, 95% CI [0.46, 1.99]; FA-Int1: Es. -1.278 ± 0.278, p-value = 0.0001, 95% CI [-2.04, -0.52]; Int1-Int2: Es. 0.941 ± 0.278, p-value = 0.007, 95% CI [0.18, 1.70]; Int1-Int3: Es. 1.114 ± 0.278, p-value = 0.0007, 95% CI [0.35, 1.70])).

EARS DOWNWARD (EAD105). The duration of the ears downward behaviour was influenced by the factors “Partner” and “Phase” (full–null model comparison: x^2^ = 53.36; df = 9; p < 0.0001). The behaviour was longer expressed towards the owner, compared to the familiar human (PartnerOWNER: Es. 1.838 ± 0.521, p-value = 0.0005, 95% CI [0.81, 2.86] – Figure 12). Overall, it was expressed more during the first and the third interactions compared to the face-away episode (FA-Int1: Es. -4.107 ± 0.816, p-value < 0.0001, 95% CI [-6.35, -1.87]; FA-Int3: Es. -2.878 ± 0.816, p-value = 0.004, 95% CI [-5.12, -0.64]) and more during the first interaction compared to the still-face as well (Int1-SF: Es. 3.008 ± 0.816, p-value = 0.0025, 95% CI [0.77, 5.25]) (Figure 13). An effect of sex was also revealed, with female dogs keeping their ears downward more than males (SexM: Es. -3.622 ± 1.126, p-value = 0.003, 95% CI [-5.92, -1.33]).

PROXIMITY: NEAR (<1mt). The full-null model comparison revealed a significant effect of the interaction between the factors “Partner”*“Phase” (full–null model comparison: x^2^ = 60.18; df = 13; p < 0.0001). Specifically, dogs stayed closer for longer time to the social partner when they were the owner (PartnerOWNER: Es. 7.490 ± 2.106, p-value = 0.0004, 95% CI [3.35, 11.6] – Figure 20), during the first interaction with the owner compared to the same with the familiar human (Int1FH-Int1OW: Es. -7.448 ± 2.11, p-value = 0.017, 95% CI [-14.20, -0.78]) and during the first interaction compared to the face-away non-interaction episode (FA-Int1: Es. -4.562 ± 1.49, p-value = 0.019, 95% CI [-8.65, -0.48]).

PROXIMITY: FAR (>1mt). The full-null model comparison revealed a significant effect of the interaction between the factors “Partner”*“Phase” (full–null model comparison: x^2^ = 64.22; df = 13; p < 0.0001).

Specifically, dogs stayed farther for longer time to the social partner when they were the familiar human (PartnerOWNER: Es. -5.778 ± 2.122, p-value = 0.007, 95% CI [-9.95, -1.60] – Figure 22), during different trial phases with the familiar human (Int1FH-Int1OW: Es. 7.435 ± 2.12, p-value = 0.018, 95% CI [0.67, 14.20]; Int3FH-Int3OW: Es. 9.166 ± 2.12, p-value = 0.0009, 95% CI [2.40, 15.93]) and during the face-away and the last interaction episodes compared to the first interaction (FA-Int1: Es. 4.736 ± 1.50, p-value = 0.015, 95% CI [0.63, 8.84]; Int1-Int3: Es. -4.342 ± 1.50, p-value = 0.032, 95% CI [-8.45, -0.24]).

HEAD SOCIAL PARTNER. The full-null model comparison revealed a significant effect of the interaction between the factors “Partner”*“Phase” (full–null model comparison: x^2^ = 85.89; df = 13; p < 0.0001). Results show a general effect of the first interaction episode, with dogs keeping their head for longer time straight towards their social partner (Int1-Int3: Es. 2.836 ± 0.89, p-value = 0.014, 95% CI [0.75, 5.65]; FA-Int1: Es. -4.830 ± 0.89, p-value < 0.0001, 95% CI [-7.28, -2.38]; Int1-SF: Es. 4.413 ± 0.89, p-value < 0.0001, 95% CI [1.96, 6.87] – Figure 27). More specifically, an effect on the social partner ID on specific phases was also found in the same interaction condition dogs kept their head for longer time towards the social partner when this was the owner (Int1FH-Int1OW: Es. -4.176 ± 1.27, p-value = 0.036, 95% CI [-8.22, -0.13]; Int2FH-Int2OW: Es. -4.049 ± 1.27, p-value = 0.049, 95% CI [-8.09, -0.01]; Int3FH-Int3OW: Es. -5.328 ± 1.27, p-value = 0.001, 95% CI [-9.37, -1.29]). Also, different phases with the same social partner elicited more or less this behaviour (FAOwner-Int1Owner: Es. -6.116 ± 1.26, p-value = 0.0001, 95% CI [-10.14, -2.09]; Int1Owner-SFOwner: Es. 4.954 ± 1.26, p-value = 0.0043, 95% CI [0.92, 8.98]).

1. ***Behaviours influenced by specific Phases***

HEAD TURNING. The frequency of the head turning behaviour (both to the left and to the right) was revealed to be influenced by the factor “Phase” (full–null model comparison: x^2^ = 58; df = 9; p < 0.0001). Dogs displayed this behaviour more frequently during the first interaction compared to all the other episodes (Int1-SF: Es. 0.423 ± 0.083, p-value < 0.0001, 95% CI [0.19, 0.65]; FA-Int1: Es. -0.476 ± 0.084, p-value < 0.0001, 95% CI [-0.70, -0.25]; Int1-Int2: Es. 0.225 ± 0.078, p-value = 0.032, 95% CI [0.01, 0.44]; Int1-Int3: Es. 0.388 ± 0.082, p-value < 0.0001, 95% CI [0.16, 0.61]) and during the second interaction compared to the face-away episode (FA-Int2: Es. -0.251 ± 0.088, p-value = 0.035, 95% CI [-0.49, -0.01]).

SNIFFING THE HUMAN PARTNER (AD40). The full-null model comparison revealed a significant effect of the “Phase” factor (full–null model comparison: x^2^ = 22.38; df = 9; p = 0.008). More specifically, this behaviour was expressed longer during the first interaction episode compared to all the others, with the exception of the still-face episode (Int1-Int2: Es. 0.926 ± 0.328, p-value = 0.040, 95% CI [0.03, 1.82]; Int1-Int3: Es. 0.928 ± 0.328, p-value = 0.039, 95% CI [0.03, 1.83]; FA-Int1: Es. -0.927 ± 0.328, p-value = 0.039, 95% CI [-1.83, -0.03]).

1. ***Effect of the control predictors on the behaviours***

Some behavioural displays were not affected by neither the testing phase, nor the social partner ID, but the analyses revealed an effect of some control predictors on their expression and/or duration.

The BLINKING (AU145) behaviour was expressed more by female dogs (SexM: Es. -0.460 ± 0.222, p-value = 0.038, 95% CI [-0.86, -0.02]) and more in the Parma facility (LocationParma: Es. -0.618 ± 0.223, p-value = 0.006, 95% CI [0.19, 1.00]).

The duration of the EARS ADDUCTOR (EAD102) behaviour was influenced by the location (LocationParma: Es. -5.783 ± 1.182, p-value < 0.0001, 95% CI [-8.17, -3.39]).

A major effect on the duration of the INNER BROW RAISER (AU101) behaviour was given by the control predictors of age, location and sex. Female dogs expressed this behaviour for longer time compared to males (SexM: Es. -2.397 ± 0.856, p-value = 0.009, 95% CI [-4.00, -0.79]), as well as older dogs compared to youngsters (Age: Es. 0.266 ± 0.114, p-value = 0.025, 95% CI [0.05, 0.48] – Figure 18). The behaviour was also significantly mostly showed in the Desenzano del Garda facility (LocationParma: Es. -3.179 ± 0.881, p-value = 0.0008, 95% CI [-4.86, -1.53]).

***Detailed results of the models including the three interaction phases (Int1, Int2, Int3) and the Social Partner (Owner, Familiar Human)***

***Behaviours in the three interaction phases***

***Table 3.*** Effect of the three interaction phases (Int1, Int2, Int3) and the identity of the social partner (in green). In light blue the behaviours whose expression decrease from Int1; in light red the behaviours increasing from Int1.

| **Behaviour** | **Social Partner**  **OWNER** | | | **Social Partner**  **FAMILIAR HUMAN** |
| --- | --- | --- | --- | --- |
| Nose-Licking (AD137) | | **Int1/Int2**  Es. 0.588 ± 0.211, p = 0.015 \| CI [0.09, 1.08] | **Int1/Int2**  ns | |
|  |  | **Int1/Int3**  Es. 0.709 ± 0.219, p = 0.003 \| CI [0.19, 1.22] | **Int1-Int3**  ns | |
|  |  | **Int2/Int3**  ns | **Int2/Int3**  ns | |
| Ear Flattener (EAD103) | | **Int1/Int2**  Es. 1.883 ± 0.479, p = 0.0004 \| CI [0.75, 3.01] | **Int1/Int2**  ns | |
|  |  | **Int1/Int3**  Es. 1.835 ± 0.479 p = 0.0005 \| CI [0.70, 2.97]) | **Int1-Int3**  ns | |
|  |  | **Int2/Int3**  ns | **Int2/Int3**  ns | |
| Ear Downward (EAD105) | | **Int1/Int2**  Es. 3.367 ± 1.24, p = 0.02 \| CI [0.43, 6.31] | **Int1/Int2**  ns | |
|  |  | **Int1-Int3**  ns | **Int1-Int3**  ns | |
|  |  | **Int2/Int3**  ns | **Int2/Int3**  ns | |
| Head Turning (AD51+AD52) | | **Int1/Int2**  Es. 0.343 ± 0.116, p = 0.009 \| CI [0.07, 0.61] | **Int1/Int2**  Es. 7.70 ± 1.77, p = 0.0001 \| CI [3.52, 11.86]; | |
|  |  | **Int1/Int3**  Es. 0.320 ± 0.115, p = 0.015 \| CI [0.05, 0.59] | **Int1-Int3**  ns | |
|  |  | **Int2/Int3**  ns | **Int2/Int3**  Es. 0.333 ± 0.120, p = 0.0154 \| CI [0.05, 0.61] | |
| Tail Wagging | | **Int1/Int2**  Es. 7.70 ± 1.77, p = 0.0001\| CI [3.52, 11.86] | **Int1/Int2**  ns | |
|  |  | **Int1/Int3**  Es. 7.81 ± 1.77, p = 0.0001 \| CI [3.63, 11.94] | **Int1/Int3**  ns | |
|  |  | **Int2/Int3**  ns | **Int2/Int3**  ns | |
| Head Human | | **Int1/Int2**  ns | **Int1/Int2**  ns | |
|  |  | **Int1/Int3**  ns | **Int1/Int3**  Es. 3.77 ± 1.33, p = 0.014 \| CI [0.64, 6.91] | |
|  |  | **Int2/Int3**  ns | **Int2/Int3**  ns | |
| Near (<1mt) | | **Int1/Int2**  ns | | |
|  |  | **Int1/Int3**  Es. 3.88 ± 1.47, p = 0.024 \| CI [0.41, 7.35] | | |
|  |  | **Int2/Int3**  ns | | |
| Far (>1mt) | | **Int1/Int2**  ns | **Int1/Int2**  ns | |
|  |  | **Int1/Int3**  ns | **Int1/Int3**  Es. -5.208 ± 2.1, p = 0.037 \| CI [-10.17, -0.25] | |
|  |  | **Int2/Int3**  ns | **Int2/Int3**  ns | |

NOSE-LICKING (AD137): The behaviour was influenced by the Interaction “Trial” and the “Partner” ID (full–null model comparison: x^2^ = 33.801; df = 5; p=2.609e-06). Dogs licked their nose more frequently during the first interaction trial with the owner compared to the other two interaction trials (OWNER - **Int1/Int3**: Es. 0.709 ± 0.219, p-value = 0.003, 95% CI [0.19, 1.22]; **Int1-Int2**: Es. 0.588 ± 0.211, p-value = 0.015, 95% CI [0.09, 1.08]). No differences were found between the interaction trials with the familiar human.

EAR FLATTENER (EAD103). The duration of the ears flattener behaviour was influenced by the factors Interaction “Trial” and the “Partner” ID (full–null model comparison: x^2^ = 21.80; df = 5; p = 0.0006). The dogs kept their ears in the flattener position longer during the first interaction trial with the owner compared to the other two interaction trials (OWNER - **Int1/Int2**: Es. 1.883 ± 0.479, p-value = 0.0004, 95% CI [0.75, 3.01]; **Int1/Int3**: Es. 1.835 ± 0.479, p-value = 0.0005, 95% CI [0.70, 2.97]). No differences emerged across the three interactions when the social partner was the familiar human.

EAR DOWNWARD (EAD105). The duration of the ears flattener behaviour was influenced by the factors Interaction “Trial” and the “Partner” ID (full–null model comparison: x^2^ = 15.80; df = 5; p = 0.007). The dogs kept their ears in the downward position longer during the first interaction trial with the owner compared to the second interaction (OWNER - **Int1/Int2**: Es. 3.367 ± 1.24, p-value = 0.020, 95% CI [0.43, 6.31]). No differences emerged across the three interactions when the social partner was the familiar human.

HEAD TURNING (AD51+AD52). The frequency of the head turning behaviour (both to the left and to the right) was revealed to be influenced by the factor “Trial” and “Partner” (full–null model comparison: x^2^ = 78.83; df = 21; p = 1.27e-08). Dogs displayed this behaviour more frequently during the first interaction compared to the two interactions trials with the owner (OWNER - **Int1/Int2**: Es. 0.343 ± 0.116, p-value = 0.009, 95% CI [0.07, 0.61]; **Int1/Int3**: Es. 0.320 ± 0.115, p-value = 0.015, 95% CI [0.05, 0.59]) and more frequently in the first and second interaction trial compared to the third with the familiar human (FAMILIAR HUMAN – **Int1/Int3**: Es. 0.457 ± 0.117, p-value = 0.0003, 95% CI [0.18, 0.73]; **Int2/Int3**: Es. 0.333 ± 0.120, p-value = 0.0154, 95% CI [0.05, 0.61]).

TAIL WAGGING. The duration of the tail wagging behaviour was influenced by the factor “Trial” and “Partner” (full–null model comparison: x^2^ = 29.71; df = 5; p = 1.68e-05). Dogs displayed this behaviour more during the first interaction compared to the two remaining interactions trials with the owner (OWNER - **Int1/Int2**: Es. 7.70 ± 1.77, p-value = 0.0001, 95% CI [3.52, 11.86]; **Int1/Int3**: Es. 7.81 ± 1.77, p-value = 0.0001, 95% CI [3.63, 11.94]).

Proximity NEAR (<1mt): The duration was influenced only by the Interaction “Trial” (no-interaction full–null model comparison: x^2^ = 37.15; df = 3; p = 4.269e-08). Dogs stayed nearer during the first interaction compared to the third (**Int1/Int3:** Es. 3.88 ± 1.47, p-value = 0.024, 95% CI [0.41, 7.35]).

HEAD HUMAN: The full-null model comparison revealed a significant effect of the interaction between the factors “Partner” and the Interaction “Trials” (full–null model comparison: x^2^ = 45.77; df = 5; p = 1.01e-08). Dogs kept their head toward the human partner less time during the last interaction trial compared to the first when the partner was the familiar human (FAMILIAR HIMAN **Int1/Int3:** Es. 3.77 ± 1.33, p-value = 0.014, 95% CI [0.64, 6.91]).

Proximity FAR (>1mt): The duration of the “staying far” from the social partner behaviour was influenced both by the Interaction “Trial” and the “Partner” ID (full–null model comparison: x^2^ = 41.97; df = 5; p = 5.962e-08). Dogs stayed farther with the familiar human during the third interaction compared to the first (FAMILIAR HUMAN - **Int1/Int3:** Es. -5.208 ± 2.1, p-value = 0.037, 95% CI [-10.17, -0.25]).

***Detailed significant results of the models including the first interaction (Int1), the two non-interaction phases (SF, FA) and their interaction with the Social Partner (Owner, Familiar Human).***

For the sake of clarity, only the significant findings described in the manuscript are shown below.

**NOSE LICK (AD137)**

**Full–null model comparison:** x² = 56.279; df = 5; p < 0.0001

| Contrast Phases \| Partner | Es ± SE | p-value | 95% CI |
| --- | --- | --- | --- |
| Int1 FAMILIAR HUMAN / OWNER | -0.829 ± 0.231 | 0.0003 | [-1.28, -0.37] |
| Still-Face FAMILIAR HUMAN / OWNER | -0.770 ± 0.382 | 0.044 | [-1.52, -0.02] |
| Contrast Partner \| Phases | **Es ± SE** | **p-value** | **95% CI** |
| OWNER Int1 / Still-Face | 1.052 ± 0.248 | 0.0001 | [0.47, 1.63] |
| OWNER Face-Away / Int1 | -0.965 ± 0.240 | 0.0002 | [-1.53, -0.40] |
| FAMILIAR HUMAN Int1 / Still-Face | 0.993 ± 0.370 | 0.02 | [0.13, 1.86] |

**LIP WIPING (AD37)**

**Full–null model comparison:** x² = 26.262; df = 5; p < 0.0001

| Contrast Phases \| Partner | Es ± SE | p-value | 95% CI |
| --- | --- | --- | --- |
| Int1 FAMILIAR HUMAN / OWNER | -1.03 ± 0.412 | 0.013 | [-1.83, -0.22] |
| Still-Face FAMILIAR HUMAN / OWNER | -2.61 ± 1.04 | 0.012 | [-4.64, -0.58] |
| Contrast Partner \| Phases | **Es ± SE** | **p-value** | **95% CI** |
| OWNER Face-Away / Int1 | -1.052 ± 0.248 | 0.0001 | [-1.86, -0.02] |

**EAR FORWARD (EAD101)**

**Full–null model comparison:** x² = 22.45; df = 5; p = 0.0005

| Contrast Phases \| Partner | Es ± SE | p-value | 95% CI |
| --- | --- | --- | --- |
| FAMILIAR HUMAN Int1 / Still-Face | -3.81 ± 1.33 | 0.013 | [-6.95, -0.66] |
| OWNER Face-Away / Int1 | 4.09 ± 1.33 | 0.007 | [0.94, 7.23] |
| Contrast Partner \| Phases | **Es ± SE** | **p-value** | **95% CI** |
| Still-Face FAMILIAR HUMAN / OWNER | 2.87 ± 1.33 | 0.033 | [0.24, 5.50] |

**EAR FLATTENER (EAD103)**

**Full–null model comparison:** x² = 28.81; df = 5; p < 0.0001

| Contrast Phases \| Partner | Es ± SE | p-value | 95% CI |
| --- | --- | --- | --- |
| OWNER Int1 / Still-Face | 1.837 ± 0.456 | 0.0002 | [0.76, 2.91] |
| OWNER Face-Away / Int1 | -1.948 ± 0.456 | 0.0001 | [-3.02, -0.87] |
| Contrast Partner \| Phases | **Es ± SE** | **p-value** | **95% CI** |
| Int1 FAMILIAR HUMAN / OWNER | -1.443 ± 0.456 | 0.002 | [-2.34, -0.54] |

**EAR DOWNWARD (EAD105)**

**Full–null model comparison:** x² = 46.12; df = 5; p < 0.0001

| Contrast Phases \| Partner | Es ± SE | p-value | 95% CI |
| --- | --- | --- | --- |
| OWNER Face-Away / Int1 | -4.989 ± 1.11 | <0.0001 | [-8.27, -2.95] |
| OWNER Int1 / Still-Face | 3.336 ± 1.11 | 0.006 | [0.86, 6.18] |
| Contrast Phases \| Partner | **Es ± SE** | **p-value** | **95% CI** |
| Int1 FAMILIAR HUMAN / OWNER | -3.592 ± 1.13 | 0.002 | [-5.82, -1.36] |
| Still-Face FAMILIAR HUMAN / OWNER | -2.567 ± 1.13 | 0.024 | [-4.80, -0.34] |

**PROXIMITY NEAR (<1mt)**

**Full–null model comparison:** x² = 32.91; df = 5; p = 3.914e-06

| Contrast Phases \| Partner | Es ± SE | p-value | 95% CI |
| --- | --- | --- | --- |
| OWNER Face-Away / Int1 | -5.512 ± 2.03 | 0.02 | [-10.32, -0.70] |
| Contrast Phases \| Partner | **Es ± SE** | **p-value** | **95% CI** |
| OWNER / FAMILIAR HUMAN | 5.47 ± 2.04 | 0.008 | [1.51, 9.43] |

**PROXIMITY FAR (>1 mt)**

**Full–null model comparison:** x² = 34.72; df = 5; p = 1.708e-06

| Contrast Phases \| Partner | Es ± SE | p-value | 95% CI |
| --- | --- | --- | --- |
| OWNER Int1 / Still-Face | -5.026 ± 2.03 | 0.037 | [-9.82, -0.23] |
| OWNER Face-Away / Int1 | 5.565 ± 2.03 | 0.018 | [0.77, 10.36] |
| Contrast Partner \| Phases | **Es ± SE** | **p-value** | **95% CI** |
| OWNER / FAMILIAR HUMAN | -5.69 ± 2.03 | 0.006 | [-9.64, -1.74] |

**HEAD HUMAN**

**Full–null model comparison:** x² = 56.88; df = 7; p = 6.3e-10

| Contrast Phases \| Partner | Es ± SE | p-value | 95% CI |
| --- | --- | --- | --- |
| OWNER Int1 / Still-Face | 4.954 ± 1.25 | 0.0003 | [1.99, 7.92] |
| OWNER Face-Away / Int1 | -6.116 ± 1.25 | <0.0001 | [-9.08, -3.15] |
| FAMILIAR HUMAN Int1 / Still-Face | 3.872 ± 1.25 | 0.007 | [0.91, 6.84] |
| FAMILIAR HUMAN Face-Away / Int1 | -3.544 ± 1.25 | 0.014 | [-6.51, -0.58] |
| Contrast Partner \| Phases | **Es ± SE** | **p-value** | **95% CI** |
| Int1 FAMILIAR HUMAN / OWNER | -4.24 ± 1.26 | 0.0009 | [-6.72, -1.76] |
| Still-Face FAMILIAR HUMAN / OWNER | -3.16 ± 1.26 | 0.013 | [-5.64, -0.67] |

**TAIL WAGGING**

**Full–null model comparison:** x² = 78.13; df = 7; p = 3.315e-14

| Contrast Phases \| Partner | Es ± SE | p-value | 95% CI |
| --- | --- | --- | --- |
| OWNER Int1 / Still-Face | 12.004 ± 1.71 | <0.0001 | [7.96, 16.05] |
| OWNER Face-Away / Int1 | -14.087 ± 1.71 | <0.0001 | [-18.13, -10.04] |
| FAMILIAR HUMAN Int1 / Still-Face | 5.522 ± 1.71 | 0.0043 | [1.48, 9.57] |
| FAMILIAR HUMAN Face-Away / Int1 | -5.773 ± 1.71 | 0.003 | [-9.82, -1.73] |
| Contrast Partner \| Phases | **Es ± SE** | **p-value** | **95% CI** |
| Int1 FAMILIAR HUMAN / OWNER | -5.77 ± 1.72 | 0.001 | [-9.16, -2.38] |

**HEAD TURNING (AD51+AD52)**

**Full–null model comparison:** x² = 77.98; df = 15; p = 1.63e-10

| Contrast Phases | Es ± SE | p-value | 95% CI |
| --- | --- | --- | --- |
| Int1 / Still-Face | 0.425 ± 0.083 | <0.0001 | [0.23, 0.62] |
| Face-Away / Int1 | -0.476 ± 0.084 | <0.0001 | [-0.67, -0.28] |

**HEAD ELSEWHERE**

**Full–null model comparison:** x² = 22.31; df = 5; p = 0.00046

| Contrast Phases | Es ± SE | p-value | 95% CI |
| --- | --- | --- | --- |
| Face-Away / Int1 | 3.86 ± 0.98 | 0.0003 | [1.55, 6.17] |

**BLINKING (AU145)**

**Full–null model comparison:** x² = 15.27; df = 5; p = 0.009

| Contrast Partner | Es ± SE | p-value | 95% CI |
| --- | --- | --- | --- |
| FAMILIAR HUMAN / OWNER | -0.221 ± 0.070 | 0.0015 | [0.08, 0.37] |

**HEAD GATE**

**Full–null model comparison:** x² = 17.553; df = 5; p = 0.0036

| Contrast Partner | Es ± SE | p-value | 95% CI |
| --- | --- | --- | --- |
| FAMILIAR HUMAN / OWNER | -2.16 ± 0.59 | 0.0004 | [-3.32, -0.99] |
